# TLR3 Activation by Zika Virus Stimulates Inflammatory Cytokine Production Which Dampens the Antiviral Response Induced by RIG-I-Like Receptors

**DOI:** 10.1128/JVI.01050-20

**Published:** 2021-04-26

**Authors:** Agnieszka Plociennikowska, Jamie Frankish, Thais Moraes, Dolores Del Prete, Franziska Kahnt, Claudio Acuna, Michal Slezak, Marco Binder, Ralf Bartenschlager

**Affiliations:** a Department of Infectious Diseases, Molecular Virology, University of Heidelberg, Heidelberg, Germany; b Division “Virus-Associated Carcinogenesis,” German Cancer Research Center (DKFZ), Heidelberg, Germany; c Research Group “Dynamics of early viral infection and the innate antiviral response,” Division “Virus-Associated Carcinogenesis,” German Cancer Research Center (DKFZ), Heidelberg, Germany; d BioMed X Innovation Center, Heidelberg, Germany; e Institute of Anatomy and Cell Biology, Heidelberg University, Heidelberg, Germany; University of Southern California

**Keywords:** IL-6, MDA5, RIG-I, SOCS3, STAT3, TLR3, ZIKV, astrocytes, cytokines, interferons

## Abstract

Infection with the Zika virus (ZIKV), a member of the *Flaviviridae* family, can cause serious neurological disorders, most notably microcephaly in newborns. In this study, we investigated the innate immune response to ZIKV infection in cells of the nervous system. In human neural progenitor cells (hNPCs), a target for ZIKV infection and likely involved in ZIKV-associated neuropathology, viral infection failed to elicit an antiviral interferon (IFN) response. However, pharmacological inhibition of Toll-like receptor 3 (TLR3) partially restored this deficit. Analogous results were obtained in human induced pluripotent stem cell (iPSC)-derived astrocytes, which are capable of mounting a strong antiviral cytokine response. There, ZIKV is sensed by both RIG-I and MDA5 and induces an IFN response as well as expression of proinflammatory cytokines, such as interleukin-6 (IL-6). Upon inhibition of TLR3, also in astrocytes the antiviral cytokine response was enhanced, whereas amounts of proinflammatory cytokines were reduced. To study the underlying mechanism, we used human epithelial cells as an easy-to-manipulate model system. We found that ZIKV is sensed in these cells by RIG-I to induce a robust IFN response and by TLR3 to trigger the expression of proinflammatory cytokines, including IL-6. ZIKV-induced upregulation of IL-6 activated the STAT3 pathway, which decreased STAT1 phosphorylation in a SOCS3-dependent manner, thus reducing the IFN response. In conclusion, we show that TLR3 activation by ZIKV suppresses IFN responses triggered by RIG-I-like receptors (RLR).

**IMPORTANCE** Zika virus (ZIKV) has a pronounced neurotropism, and infections with this virus can cause serious neurological disorders, most notably microcephaly and Guillain-Barré syndrome. Our studies reveal that during ZIKV infection, recognition of viral RNA by TLR3 enhances the production of inflammatory cytokines and suppresses the interferon response triggered by RIG-I-like receptors (RLR) in a SOCS3-dependent manner, thus facilitating virus replication. The discovery of this cross talk between antiviral (RLR) and inflammatory (TLR) responses may have important implications for our understanding of ZIKV-induced pathogenesis.

## Abstract (precis)

Zika virus (ZIKV) has a pronounced neurotropism, and infections with this virus can cause serious neurological disorders, most notably microcephaly and Guillain-Barré syndrome. Our studies reveal that during ZIKV infection, recognition of viral RNA by TLR3 enhances the production of inflammatory cytokines and suppresses the interferon response triggered by RIG-I-like receptors (RLR) in a SOCS3-dependent manner, thus facilitating virus replication.

## INTRODUCTION

Zika virus (ZIKV), a mosquito-borne flavivirus, received its name after the first isolation from a rhesus monkey in the Zika Forest of Uganda in 1947 (1). Until the 1980s only sporadic cases of human infections with ZIKV were reported, and the virus was associated with a mild illness and low hospitalization rates (2). However, in 2013-2014 the virus caused a large outbreak in French Polynesia concomitant with a dramatic increase in the incidence of Guillain-Barré syndrome and severe neurological complications (3). With the subsequent entrance and dissemination of ZIKV in the Americas, in late 2014 Brazil reported a remarkable increase of microcephalic cases in newborns born to ZIKV-infected mothers (reviewed in reference 4). Thereafter, the virus spread in South, Central, and North America as well as the Caribbean and was considered an emerging infectious disease. Although ZIKV circulation in these countries declined, in late 2018 the first outbreaks were reported in India (5, 6).

ZIKV is transmitted through the bite of an infected mosquito. In the epidermis and dermis, highly permissive skin fibroblasts and epidermal keratinocytes become infected (7). Subsequent transmission to immature dendritic cells (Langerhans cells) likely facilitates virus dissemination to different organs (7). In pregnant women, transplacental transmission of ZIKV to the fetus may occur through the infection of cytotrophoblasts and Hofbauer cells (8). Studies conducted in cultured human neural progenitor cells (hNPCs) (9), organoids (10), brain slices (11), and mouse brain (12) suggest that after passage of the human fetal-placental barrier, ZIKV preferentially targets NPCs in the developing central nervous system (CNS), arresting the cell cycle and cellular differentiation and triggering apoptosis (9, 10, 13, 14).

The innate immune system plays a crucial role in combating viral infections in mammalian cells. Cell-intrinsic antiviral signaling cascades begin with the detection of viral components, most importantly genomic material, acting as a pathogen-associated molecular pattern (PAMP) sensed by pattern recognition receptors (PRRs). Recognition of viral RNA is mainly mediated by a family of cytoplasmic retinoic acid-inducible gene I (RIG-I)-like receptors, comprising RIG-I, melanoma differentiation-associated gene 5 (MDA5), and laboratory of genetics and physiology 2 (LGP2). Upon binding of viral RNA, these PRRs trigger downstream a signaling cascade via the central adapter mitochondrial antiviral-signaling protein (MAVS). Another family of PRRs detecting viral nucleic acids is Toll-like receptors (TLRs). Among them, endosomal TLR3, TLR7, and TLR8 recognize double-stranded (TLR3) or single-stranded (TLR7/8) RNA. For both families of receptors, downstream signaling leads to the activation of two transcription factors: NF-κB, leading to the production of proinflammatory cytokines, and members of the interferon (IFN) regulatory factor (IRF) family inducing an antiviral response that is characterized by the production of IFNs and expression of IFN-stimulated genes (ISGs) (15–19).

Although earlier reports have shown that RIG-I is the main sensor of ZIKV RNA in epithelial cells and in HEK293T cells overexpressing RIG-I (20, 21), others proposed a joint action of RIG-I and MDA5 in skin fibroblasts (7) and in human trophoblasts (22). The role of TLR3 during ZIKV infection remains even more elusive. On the one hand, silencing of TLR3 in skin fibroblasts increases virus replication, without affecting IFN mRNA expression (7); on the other hand, disrupting TLR3 signaling in primary human astrocytes decreases ZIKV replication and dampens immune response (23). Moreover, pharmacological inhibition of TLR3 during ZIKV infection of cerebral organoids attenuates virus-mediated apoptosis and neurosphere shrinkage (24).

The goal of the present study was to characterize the innate immune response to ZIKV, especially in cells of the CNS. Focusing on hNPCs and induced pluripotent stem cell (iPSC)-derived human astrocytes, we studied the roles of RIG-I, MDA5, and TLR3 in sensing ZIKV infection and in inducing inflammatory as well as antiviral cytokines. Mechanistic follow-up experiments with a human epithelial cell model argued for a dichotomous role of TLR3 that dampens the IFN response in a suppressor of cytokine signaling 3 (SOCS3)-dependent manner while increasing the proinflammatory cytokine response.

## RESULTS

### ZIKV mounts a negligible IFN response in neural progenitor cells.

**FIG 1:**
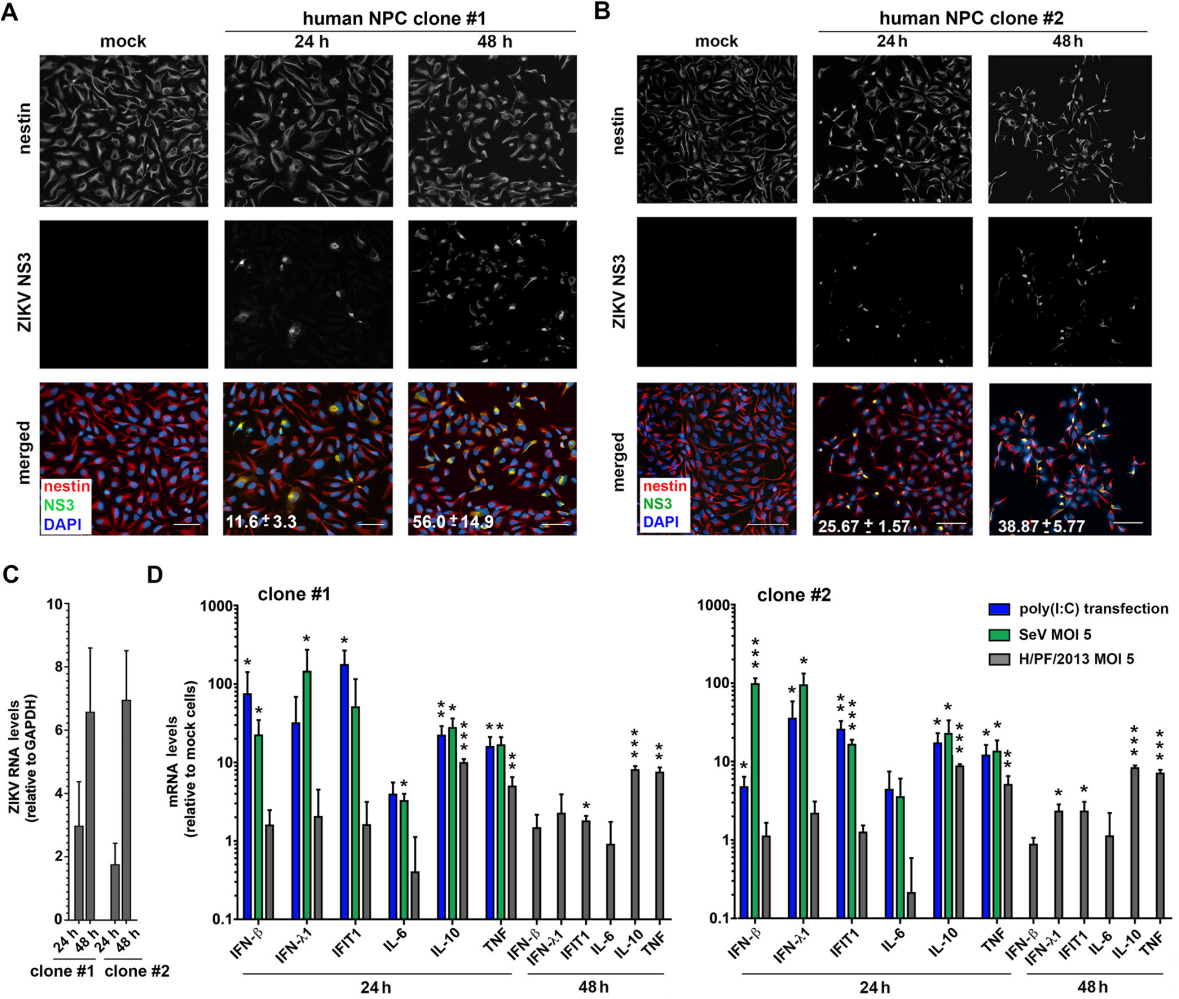
ZIKV replicates efficiently in human neural progenitor cells but fails to induce an interferon response. (A and B) hNPCs obtained from two different donors were infected with ZIKV (strain H/PF/2013; multiplicity of infection [MOI] = 5), fixed at the indicated time points, and stained for the neural marker nestin (red) and ZIKV NS3 (green). Nuclear DNA was stained with DAPI. For visualization purposes, Gamma correction was adjusted to a value of 1.5. Scale bar, 100 μm. Numbers given on the images in the bottom row correspond to the percentage of infected (NS3-positive) cells for each given condition. Shown are mean values ± SD from two independent experiments. (C) hNPCs infected as in panels A and B were lysed 24 and 48 h postinfection. Total RNA was extracted, and viral RNA was quantified by RT-qPCR. Values were normalized to GAPDH. (D) Cells of both clones were infected with ZIKV strain H/PF/2013 using an MOI of 5, infected with Sendai virus (SeV; MOI = 5), or transfected with 100 ng/ml of poly(I·C). Cells were lysed 24 and 48 h later, total RNA was extracted, and amounts of mRNAs specified at the bottom were determined by RT-qPCR. Values were normalized to GAPDH and to the respective mock sample. Data represent the mean values ± SD from three independent experiments. Statistical significance, relative to mock cells, was assessed by unpaired *t* test with Welch’s correction. ***, *P* ≤ 0.001; **, *P* ≤ 0.01; *, *P* ≤ 0.05.

With the aim to study innate immune responses to ZIKV in CNS cells, we focused on hNPCs, which are one of the most relevant cell types for ZIKV pathogenesis. To this end, we used two different cell clones derived from two different donors in order to exclude clone-specific effects. Stemness of the cells was confirmed by immunofluorescent staining of the neural stem cell marker nestin (Fig. 1A and B). Upon infection of hNPCs with ZIKV strain H/PF/2013, we observed productive replication and spread, illustrated by increases of ZIKV NS3-expressing cells 24 and 48 h postinfection (Fig. 1A and B), The time-dependent increase of intracellular viral RNA corroborated efficient virus replication (Fig. 1C). We note that longer infection times resulted in considerable cell death and therefore were not analyzed (data not shown).

To examine whether infected hNPCs are able to induce an IFN response and the transcription of ISGs, we utilized reverse transcription-quantitative PCR (RT-qPCR). However, upon ZIKV infection, in most cases the transcription of IFN-β and IFN-λ1 was not significantly upregulated in both hNPC cell clones. Only a marginal increase of the ISG IFIT1 was detected in both clones, and a slight upregulation of IFN-λ1 was detected in one of the two cell clones at 48 h postinfection (Fig. 1D). On contrast, we observed a significant increase of the inflammatory cytokines interleukin-10 (IL-10) and tumor necrosis factor (TNF), but not IL-6, already at 24 h postinfection (Fig. 1D). To exclude that hNPCs are incapable of mounting an efficient antiviral response in general, we infected the cells with the well-established model virus Sendai virus (SeV; preferential activation of RIG-I) or transfected the cells with the viral double-stranded RNA (dsRNA) mimic poly(I·C). Of note, under both conditions, except for IL-6, all tested cytokines and the ISG IFIT1 were induced (Fig. 1D). Therefore, we conclude that hNPCs are immunocompetent cells able to respond to different stimuli but do not mount a significant IFN response upon ZIKV infection.

### IFN activation in neural progenitor cells is impaired by negative cross talk between TLR3 and RLR signaling.

**FIG 2:**
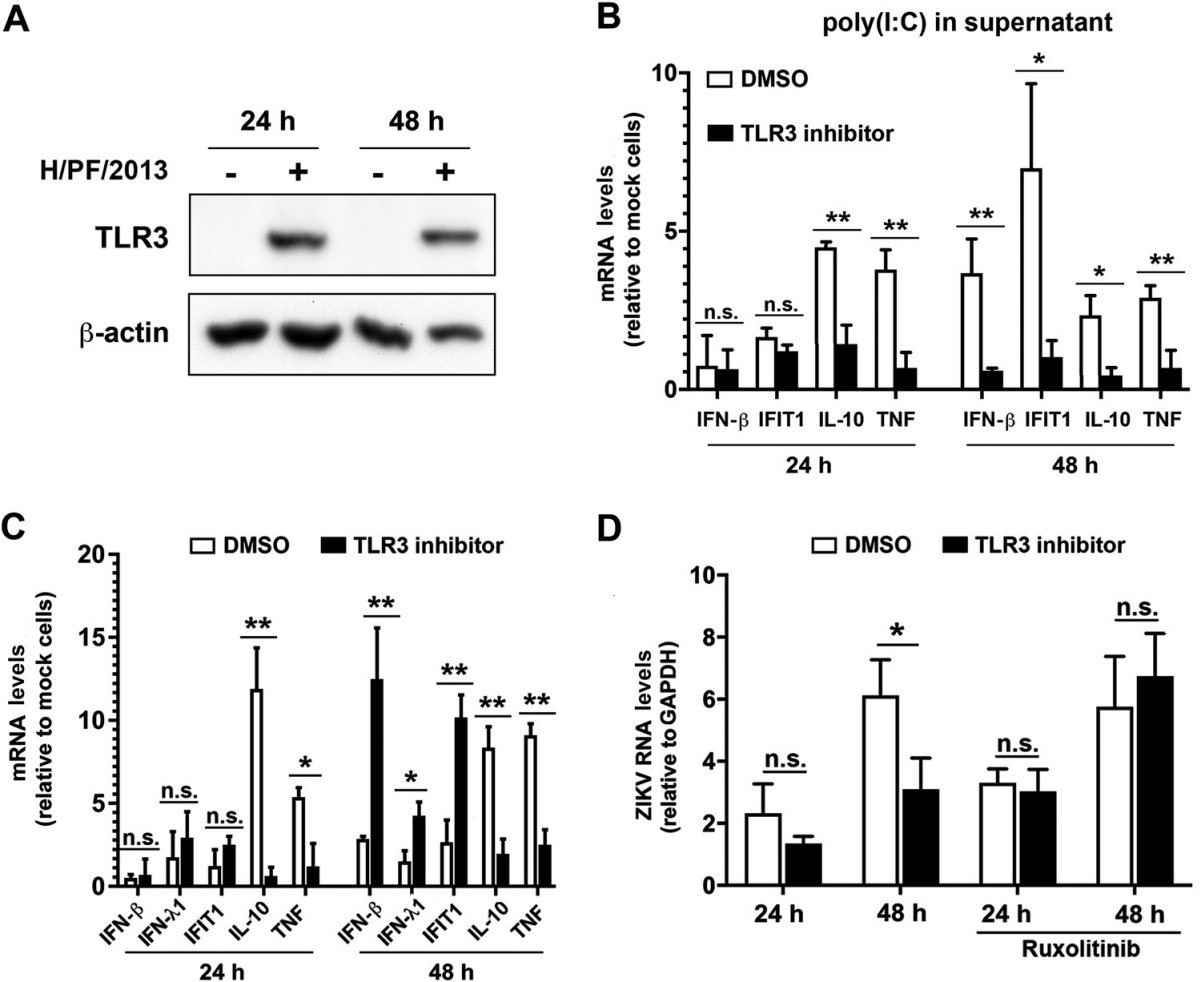
TLR3 inhibition reinstates the antiviral response against ZIKV in human neural progenitor cells. (A) Expression of TLR3 in hNPCs without or with ZIKV infection (strain H/PF/2013; MOI = 5) was determined 24 and 48 h after infection by using Western blotting. β-Actin served as the loading control. (B and C) hNPCs were incubated for 1 h with DMSO (control) or a 25 μM concentration of the TLR3/dsRNA complex inhibitor thiophene-carboxamidopropionate, followed by addition of poly(I·C) into the cell culture supernatant (50 μg/ml) (B) or ZIKV infection (strain H/PF/2013; MOI = 5) (C). After 24 and 48 h, cells were lysed, total RNA was extracted, and amounts of cytokine mRNA were determined. Values were normalized to GAPDH and the respective mock sample. (D) The JAK inhibitor ruxolitinib was added to the cell culture supernatant 2 h prior to infection at a final concentration of 1 μM. ZIKV RNA was quantified by RT-qPCR and normalized to GAPDH. The means ± SD from three independent experiments are shown. Statistical significance was assessed by unpaired *t* test with Welch’s correction. n.s., not significant. **, *P* ≤ 0.01; *, *P* ≤ 0.05.

The lack of an efficient antiviral response to ZIKV in hNPCs might be due to a specific inability of the cells to sense the virus or to an active dampening of PRR signaling. As hNPCs are capable of mounting an efficient innate immune response in general and express inflammatory cytokines upon ZIKV infection, we hypothesized that TLR3 signaling might be involved in suppressing antiviral responses induced via the RIG-I-like receptor (RLR) pathways. This assumption was based on earlier studies reporting a dual and opposed activation of TLR3 and the IFN signaling pathway (25–27). In the initial set of experiments, we determined expression of TLR3 in clone 1 of hNPCs. In agreement with earlier studies showing an upregulation of TLR3 in response to poly(I·C) (28) or bacterial and viral infection (29, 30), TLR3 was detected in hNPCs upon ZIKV infection (Fig. 2A). However, mechanistic studies in these cells by RNA interference-based approaches were flawed because transfection of small interfering RNAs (siRNAs) induced an IFN response. Therefore, as an alternative, we utilized a commercially available inhibitor of the dsRNA/TLR3 complex, thiophene-carboxamidopropionate (31), which was applied at concentrations which do not affect viability of used cells as deduced from an unaltered level of a housekeeping gene (data not shown) and in line with published literature (31). To validate inhibitor activity in hNPCs, cells were stimulated with 50 μg/ml of poly(I·C), which was added to the cell culture supernatant in order to activate TLR3 signaling. Upon inhibitor treatment, we observed a decline of IFN-β, IFIT1, IL-10, and TNF mRNA amounts, demonstrating functionality of the inhibitor (Fig. 2B). Next, we infected the cells with ZIKV strain H/PF/2013 and confirmed also in this setting a substantially decreased expression of the inflammatory cytokines IL-10 and TNF upon TLR3 inhibitor treatment. Strikingly, at the same time, we observed a significant induction of IFN-β, IFN-λ1, and IFIT1 expression 48 h postinfection (Fig. 2C). This increased expression of antiviral genes correlated with reduced ZIKV RNA replication at late time point after infection that could be restored upon treatment of cells with ruxolitinib, an inhibitor of JAK/STAT signaling (Fig. 2D). These results suggest that in hNPCs, TLR3 contributes to ZIKV-induced synthesis of inflammatory cytokines and represses the production of the IFN response, thus increasing viral replication. Moreover, the loss of replication reduction with cells treated with the TLR3 inhibitor and ruxolitinib suggests that the antiviral effect is exerted by the paracrine effect of IFN released from infected cells. While noninfected cells are protected by this IFN, owing to STAT1 degradation in infected cells, they have lost IFN responsiveness (32).

### ZIKV mounts a robust antiviral response in human astrocytes in a RIG-I and MDA5-dependent manner.

**FIG 3:**
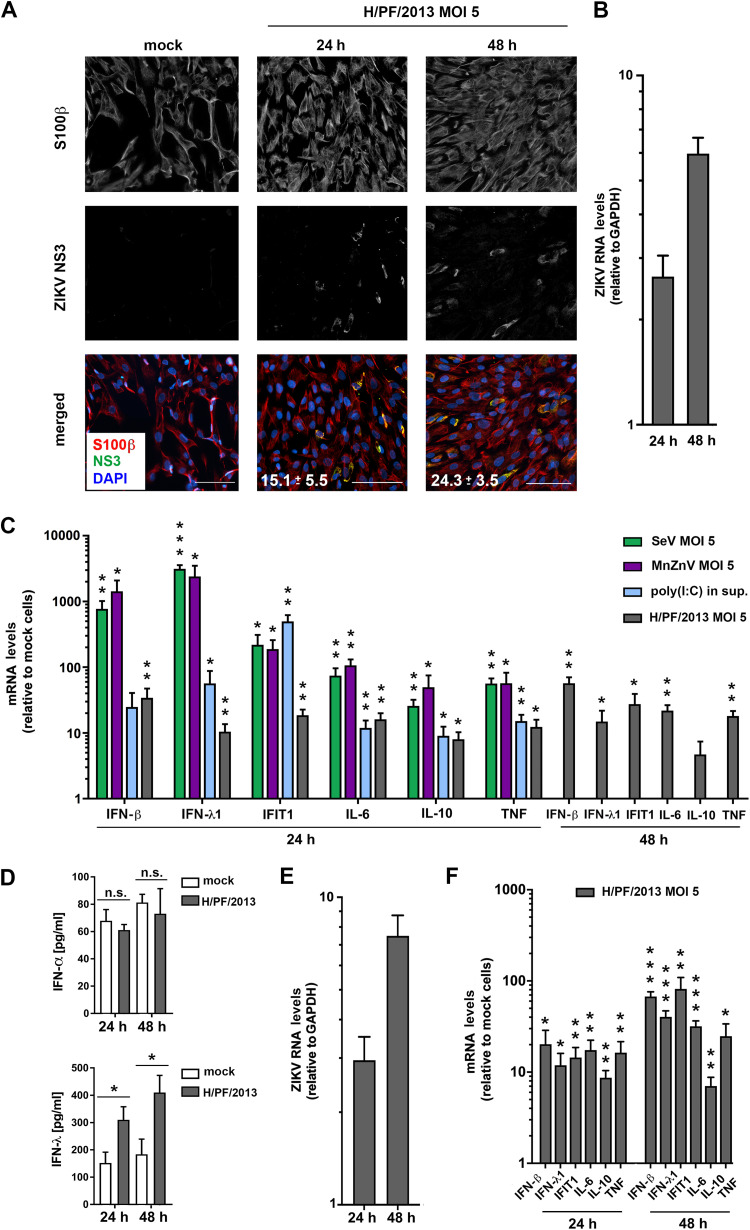
ZIKV replication in astrocytes induces a robust interferon response. (A to C) Human iPSC-derived astrocytes were infected with ZIKV strain H/PF/2013 and harvested 24 and 48 h later. (A) ZIKV spread in astrocyte cultures. Infected cells were fixed at given time points and stained for the astrocyte marker S100β and for ZIKV NS3. Nuclear DNA was stained with DAPI. For visualization purposes, Gamma correction was adjusted to a value of 1.5. Scale bar, 100 μm. Numbers given on the images in the bottom row correspond to the percentage of infected, i.e., NS3-positive, cells for each given condition. Numbers are the means ± SD from two independent experiments. (B) Total RNA was extracted from cell lysates and viral RNA was quantified by RT-qPCR. Values were normalized to GAPDH. (C) Astrocytes were infected with SeV (MOI = 5) or MnZnV (MOI = 5) or stimulated with 10 μg/ml of poly(I·C) that was added into the culture supernatant. Cells were lysed and amounts of mRNAs specified at the bottom were determined by RT-qPCR. Values were normalized to GAPDH and to the respective mock sample that was set to 1. Mean values ± SD from three independent experiments are shown. (D) Amounts of IFN-α and IFN-λ released into culture supernatants were quantified by ELISA. Shown are the mean values ± SD from two independent experiments. (E and F) iPSC-derived astrocytes generated with different differentiation protocols were infected with ZIKV and analyzed like for panels B and C. Mean values ± SD from two independent experiments are shown. For panels C and F, statistical significance was assessed by unpaired *t* test with Welch’s correction. ***, *P* ≤ 0.001; **, *P* ≤ 0.01; *, *P* ≤ 0.05.

To gain further insight into the innate immune response to ZIKV in the nervous system, we turned to astroglial cells, shown previously to mount an IFN response against ZIKV (33, 34). We used astrocytes differentiated from a human iPSC line and confirmed their differentiation state by immunofluorescent staining of the astrocyte marker S100β (Fig. 3A). Cells were infected with ZIKV (strain H/PF/2013), revealing the highest number of ZIKV-positive cells (∼24%) at 48 h postinfection (Fig. 3A). Similar to the case with hNPCs, time points above 48 h were not considered due to cell death (data not shown). Efficient ZIKV replication was corroborated by quantification of intracellular viral RNA using RT-qPCR (Fig. 3B). To confirm functional RLR and TLR3 pathways of used astrocytes, we infected them with SeV (RIG-I) or MnZnV (MDA5) or stimulated the cells with 10 μg/ml of poly(I·C) added to the cell culture supernatant for 24 h to activate TLR3. Upon all three stimuli, the astrocytes mounted a robust cytokine response (Fig. 3C). Of note, infection of these cells with ZIKV also induced transcription of IFN, IFIT1, and inflammatory cytokine genes (Fig. 3C). To corroborate these data, we measured IFNs released into cell culture supernatants by using enzyme-linked immunosorbent assay (ELISA). While IFN-β levels were below the detection limit (data not shown) and amounts of IFN-α remained unchanged, IFN-λ levels were elevated in supernatants of cells infected with ZIKV compared to those of mock-inoculated cells (Fig. 3D).

To exclude effects possibly caused by the protocol used to differentiate astrocytes, we generated astrocytes with another differentiation method (see Materials and Methods). ZIKV infection of these astrocytes led to very similar results with respect to viral replication dynamics (Fig. 3E) and cytokine response (Fig. 3F).

**FIG 4:**
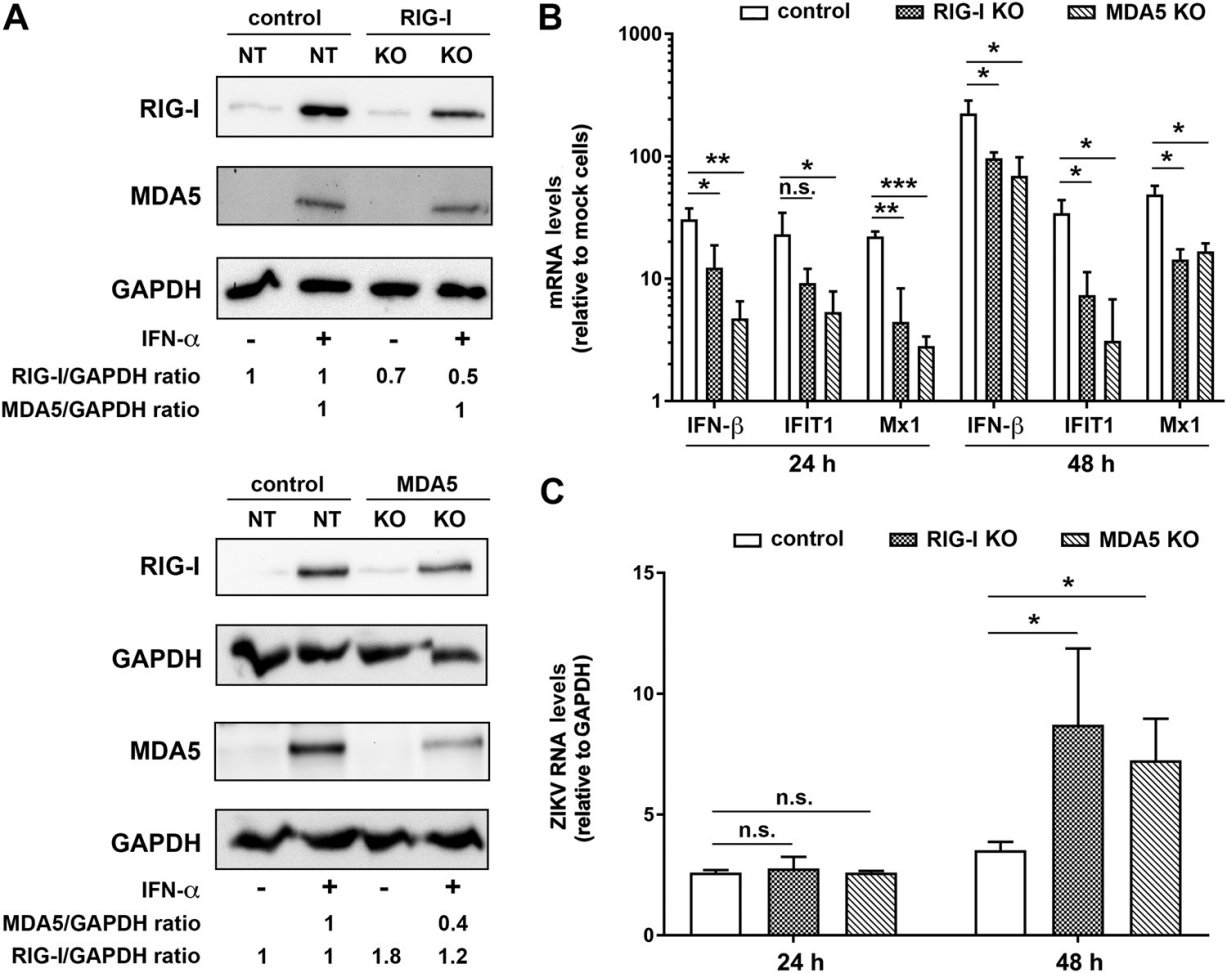
ZIKV induces an antiviral response in astrocytes in a RIG-I- and MDA5-dependent manner. RIG-I and MDA5 were depleted from human iPSC-derived astrocytes using CRISPR*/*Cas9-mediated KO of either PRR. Nontargeting (NT) gRNA was used as a control. Cell pools were used for all analyses. (A) Protein levels of RIG-I and MDA5 were determined by Western blotting using lysates of untreated cells and cells treated with 250 U/ml of IFN-α to boost RIG-I and MDA5 expression. GAPDH was used as a loading control. Numbers below the lanes refer to protein amounts normalized to GAPDH and to control cells, which were set to 1. (B and C) Cells were infected with ZIKV (strain H/PF/2013; MOI = 5), and samples were collected 24 and 48 h postinfection. Total RNA was extracted from cell lysates, and RNA amounts were quantified by RT-qPCR using IFN-β, IFIT1, and Mx1-specific primers (B) or ZIKV-specific primers and a TaqMan probe (C). Values were normalized to GAPDH and in panel B are expressed as fold of mock cells. Shown are the mean values ± SD from three (24 h) and two (48 h) independent experiments. Statistical significance was assessed by unpaired *t* test with Welch’s correction. ***, *P* ≤ 0.001; **, *P* ≤ 0.01; *, *P* ≤ 0.05.

With the aim to determine the PRR sensing ZIKV in human iPSC-derived astrocytes, we reduced the levels of endogenous RIG-I and MDA5 by siRNA-mediated knockdown. However, we found the siRNA itself to be immunostimulatory in these cells (a phenomenon widely discussed more recently in reference 35). To overcome this problem, we employed a CRISPR/Cas9-based knockout (KO) approach for RIG-I and MDA5 depletion that was validated in the cell pools by treating them with IFN-α to induce expression of the two PRRs (themselves being ISGs). Western blot analysis confirmed reduction of RIG-I and MDA5 levels by ∼50% (Fig. 4A, upper and lower graphs, respectively). Upon infection of these cells with ZIKV for 24 and 48 h, we observed a decreased antiviral response, as evidenced by significantly lower mRNA levels of IFN-β and the ISGs IFIT1 and Mx1 (Fig. 4B). Reduced IFN response correlated with an increase of ZIKV RNA amount in both KO cell pools at 48 h postinfection (Fig. 4C). These data suggest that both RIG-I and MDA5 are involved in ZIKV-induced antiviral responses in human iPSC-derived astrocytes.

### TLR3 activation by ZIKV in human astrocytes promotes the expression of proinflammatory cytokines and dampens the antiviral response.

**FIG 5:**
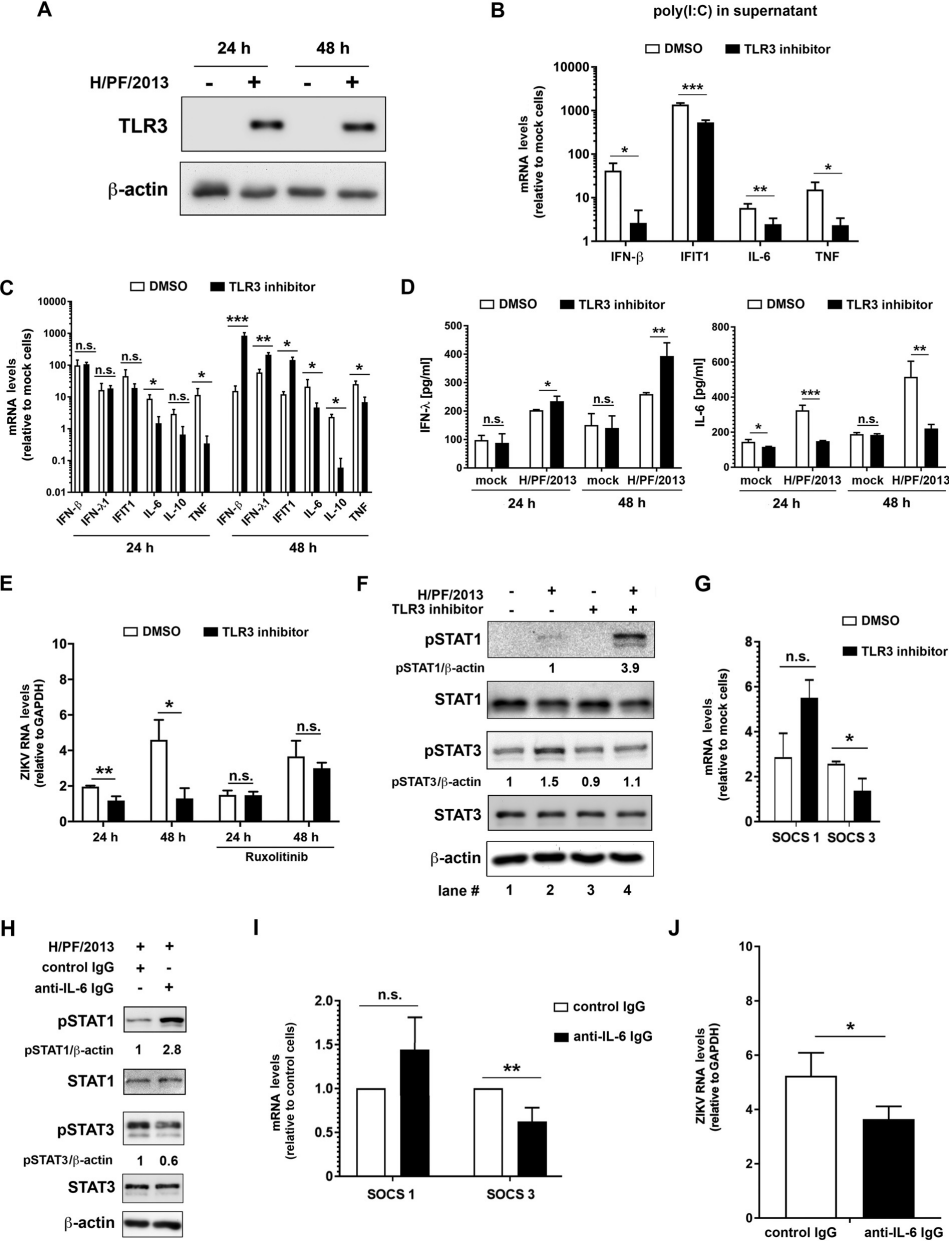
TLR3 inhibition in human astrocytes increases the antiviral response to ZIKV and dampens the production of inflammatory cytokines. (A) Expression of TLR3 in human iPSC-derived astrocytes was determined 24 and 48 h after ZIKV infection (strain H/PF/2013; MOI = 5) or mock infection by using Western blotting. β-Actin served as a loading control. (B to G) Astrocytes were incubated for 1 h with DMSO (control) or a 25 μM concentration of the TLR3/dsRNA complex inhibitor thiophene-carboxamidopropionate prior to addition of poly(I·C) into the cell culture medium (10 μg/ml) or ZIKV infection (strain H/PF/2013; MOI = 5). (B) After 24 h, total RNA was extracted, and amounts of mRNAs specified at the bottom were determined by RT-qPCR. (C) Astrocytes were infected with ZIKV and 24 and 48 h later, cells were lysed and IFN-β, IFN-λ1, IFIT1, IL-6, IL-10, and TNF mRNA amounts were quantified by RT-qPCR. Values were normalized to GAPDH and the respective mock sample. (D) IFN-λ and IL-6 released into culture supernatants of infected cells were quantified by ELISA. (E) Cells from the same experiment were used to quantify viral RNA by RT-qPCR using a TaqMan probe. Ruxolitinib was used as described for Fig. 2D and values were normalized to GAPDH. (F) Protein levels of phosphorylated and total STAT1 and STAT3 were determined by Western blotting using lysates of cells infected for 48 h. β-Actin was used as a loading control. Numbers below the lanes refer to protein amounts normalized to β-actin and to control cells, which were set to 1. (G) At 48 h after infection, total RNA was extracted and mRNA amounts of SOCS1 and SOCS3 were determined by RT-qPCR. Values were normalized to GAPDH and to the respective mock sample. (H to J) Astrocytes were infected with ZIKV in the presence of 5 μg/ml of anti-IL-6 IgG or control IgG for 48 h. (H) Expression of STATs was determined by Western blotting. Numbers below the lanes refer to protein amounts normalized to β-actin and to control cells, which were set to 1. (I and J) Total RNA was extracted from cell lysates, and RNA amounts were quantified by RT-qPCR using SOCS1- and SOCS3-specific primers (I) or ZIKV-specific primers and a TaqMan probe (J). Values in panel I were normalized to GAPDH and are expressed as fold of control cells, which were set to 1. In both panels, the mean values ± SD from three independent experiments are shown. Statistical significance was assessed by unpaired *t* test with Welch’s correction. ***, *P* ≤ 0.001; **, *P* ≤ 0.01; *, *P* ≤ 0.05.

To corroborate the negative effect of TLR3 on the antiviral response to ZIKV infection also in human iPSC-derived astrocytes, which express TLR3 in an inducible manner like hNPCs (Fig. 5A and 2A, respectively), we made use of the dsRNA/TLR3 complex inhibitor. The efficacy of this compound in astrocytes was confirmed by treatment of the cells with 10 μg/ml of poly(I·C) added into the culture medium in the presence of the inhibitor or, as a control, in cells treated with dimethyl sulfoxide (DMSO). Inhibitor treatment significantly decreased the expression of both antiviral (IFN-β) and proinflammatory (IL-6 and TNF) cytokines as well as the ISG IFIT1 (Fig. 5B). We then infected the astrocytes with ZIKV and observed profound reduction of the proinflammatory cytokines IL-6, IL-10, and TNF and a striking ∼50-fold, ∼3.5-fold, and ∼10-fold increase of IFN-β, IFN-λ1, and IFIT1 mRNA, respectively, at 48 h postinfection (Fig. 5C). Changes in mRNA levels upon inhibitor treatment correlated well with IFN-λ and IL-6 protein levels in culture supernatants (Fig. 5D). A consistent reduction of intracellular ZIKV RNA abundance was detected in cells treated with the TLR3 inhibitor, which was reinstated by inhibition of IFN signaling with ruxolitinib (Fig. 5E), similar to what we observed with hNPCs (Fig. 2D).

To examine these changes in antiviral and inflammatory cytokine activation further, we investigated phosphorylation of STAT1 and STAT3, which are involved in IFN- and IL-6-induced signaling, respectively (reviewed in reference 36). In addition, we monitored the expression of suppressor of cytokine signaling (SOCS) proteins that are induced upon STAT1 and STAT3 activation. Upon ZIKV infection of iPSC-derived astrocytes, we observed an almost 4-fold increase of pSTAT1 in cells treated with the TLR3 inhibitor (Fig. 5F, top blot, lane 4), whereas phosphorylation of STAT3 was decreased to the level of noninfected cells (Fig. 5F, compare lane 2 with lane 4). Consistent with this result, mRNA levels of SOCS3 were significantly lower than in control cells (Fig. 5G).

Since members of the IL-6-type cytokine family are well-known activators of STAT3, which induces SOCS3 expression (reviewed in references 37 and 38), we examined whether blocking IL-6 signaling could phenocopy TLR3 inhibition. Indeed, ZIKV infection under conditions of antibody-mediated sequestration of IL-6, which was released from the cells into the culture supernatant, resulted in ∼3-fold-enhanced STAT1 phosphorylation (Fig. 5H, top blot), whereas STAT3 phosphorylation (Fig. 5H) and SOCS3 expression (Fig. 5I) were significantly reduced. Enhanced IFN signaling, indicated by elevated pSTAT1 levels and achieved by antibody-mediated IL-6 sequestration (Fig. 5H, top blot), correlated with reduced RNA replication of ZIKV (Fig. 5J). Taken together, our data suggest that TLR3 activation by ZIKV plays an important role in dampening antiviral responses in cells of the human CNS (hNPCs and astrocytes), possibly via promoting the expression of proinflammatory cytokines.

### TLR3-mediated reduction of the antiviral response and increase of the inflammatory cytokine response also in the A549 human epithelial cell model.

**FIG 6:**
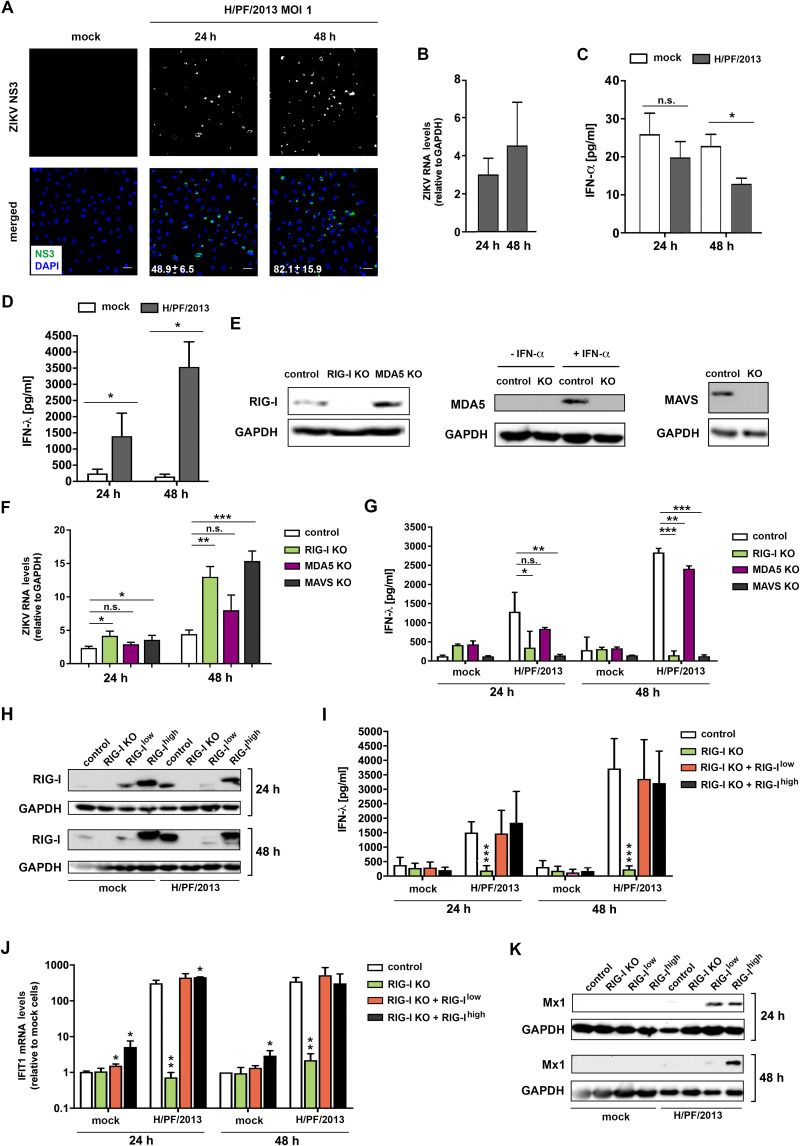
ZIKV replication in A549 cells induces an interferon response in a RIG-I-dependent manner. (A) A549 cells were infected with ZIKV strain H/PF/2013 (MOI = 1). Cells were fixed 24 and 48 h postinfection and stained for ZIKV NS3. Nuclear DNA was stained with DAPI. For visualization purposes, Gamma correction was adjusted to a value of 1.5. Scale bar, 100 μm. Numbers given on the images in the bottom row correspond to the percentage of infected cells and are the means ± SD from two independent experiments. (B) Viral RNA was quantified by RT-qPCR using a TaqMan probe. Values were normalized to GAPDH. (C and D) IFN-α (C) and IFN-λ (D) released into culture supernatants were quantified by ELISA. Data represent the mean values ± SD from three independent experiments. (E) Protein abundances of RIG-I, MDA5, and MAVS in A549-derived RIG-I KO, MDA5 KO, and MAVS KO cells were determined by Western blotting. A549 MDA5 KO cells were treated with 100 U/ml of IFN-α to confirm the absence of the protein under MDA5-inducing conditions (middle). GAPDH was used as a loading control. (F and G) RIG-I is the primary sensor of ZIKV in human epithelial-like cells. A549 control cells as well as RIG-I KO, MDA5 KO, and MAVS KO cells were infected with ZIKV strain H/PF/2013 (MOI = 1). Samples were collected 24 and 48 h postinfection. (F) Total RNA was extracted from the cell lysates and viral RNA was quantified by RT-qPCR using ZIKV-specific primers and a TaqMan probe. Values were normalized to GAPDH. (G) Amounts of IFN-λ released into the supernatants of infected cells were quantified by ELISA. Statistical significance was assessed by unpaired *t* test with Welch’s correction. ***, *P* ≤ 0.001; **, *P* ≤ 0.01; *, *P* ≤ 0.05. (H to K) RIG-I reconstitution in A549 KO cells restores the innate immune response against ZIKV. Control A549 cells (control) and A549 RIG-I KO and A549 RIG-I-KO cells reconstituted to express low or high levels of RIG-I by using expression constructs with different promoters were infected with ZIKV (strain H/PF/2013; MOI = 1). Samples were collected 24 and 48 h postinfection. (H) RIG-I abundance in cells specified above each lane by using Western blotting. GAPDH was used as a loading control. (I) IFN-λ released into culture supernatants of infected cells was quantified by ELISA. (J) Total RNA was extracted from cell lysates, and abundance of IFIT1 mRNA was determined by RT-qPCR. Values were normalized to GAPDH and are expressed relative to values of mock cells. (K) Mx1 protein levels were determined by Western blotting. GAPDH served as a loading control. Data in panels I and J represent the mean values ± SD from three independent experiments. Statistical significance was assessed by unpaired *t* test with Welch’s correction for RIG-I KO and reconstituted cells, compared to the respective control cells for each condition and time point. ***, *P* ≤ 0.001; **, *P* ≤ 0.01; *, *P* ≤ 0.05.

Mechanistic studies on the dichotomous role of TLR3 in antiviral and proinflammatory cytokine responses were not possible in hNPCs and iPSC-derived human astrocytes because of limited access to these cells and their high sensitivity to mount cytokine responses upon experimental manipulation. Therefore, we resorted to the well-established human epithelial cell line model A549, which is easy to handle yet mounts robust cytokine responses upon viral infection (21, 39). In the initial set of experiments, A549 cells were infected with ZIKV (strain H/PF/2013, as used for all previous experiments) and efficient infection and replication were validated by immunofluorescence and quantification of intracellular viral RNA (Fig. 6A and B). Since IFN-β levels were below the detection limit (data not shown) and amounts of IFN-α were only very moderately affected, similar to what we found for astrocytes (compared Fig. 6C with Fig. 3D), we focused on type III IFN and observed high levels of IFN-λ released into the culture supernatant of ZIKV-infected cells (Fig. 6D).

To determine the PRR responsible for sensing of ZIKV in this cell line, we generated A549 KO cells lacking RIG-I, MDA5, or MAVS (Fig. 6E). We found that ablation of the RIG-I gene increased replication of ZIKV ∼2- and 3-fold at 24 and 48 h postinfection, respectively, as determined by quantification of intracellular viral RNA (Fig. 6F). At 48 h postinfection, a comparable increase in ZIKV RNA was observed in cells depleted of MAVS, whereas in cells lacking MDA5, a statistically insignificant trend was found (Fig. 6F). To correlate viral replication with antiviral IFN response, we measured the production of IFN-λ detectable in culture supernatants. Consistent with the replication data, we found that RIG-I KO cells and cells lacking MAVS produced virtually no IFN-λ upon ZIKV infection (Fig. 6G). In contrast, MDA5 KO cells were still able to mount a robust response (Fig. 6G). We corroborated these findings by using RIG-I KO cells, reconstituted with functional RIG-I that was transcribed under the control of two different promoters, allowing for low and high expression of the gene (Fig. 6H). Upon ZIKV infection, IFN-λ and the ISGs IFIT1 and Mx1 were selectively induced in the reconstituted cells, thus excluding off-target effects of the RIG-I KO (Fig. 6I to K). Taken together, these findings show that RIG-I is the main sensor for ZIKV in A549 cells, consistent with a recent report (21).

**FIG 7:**
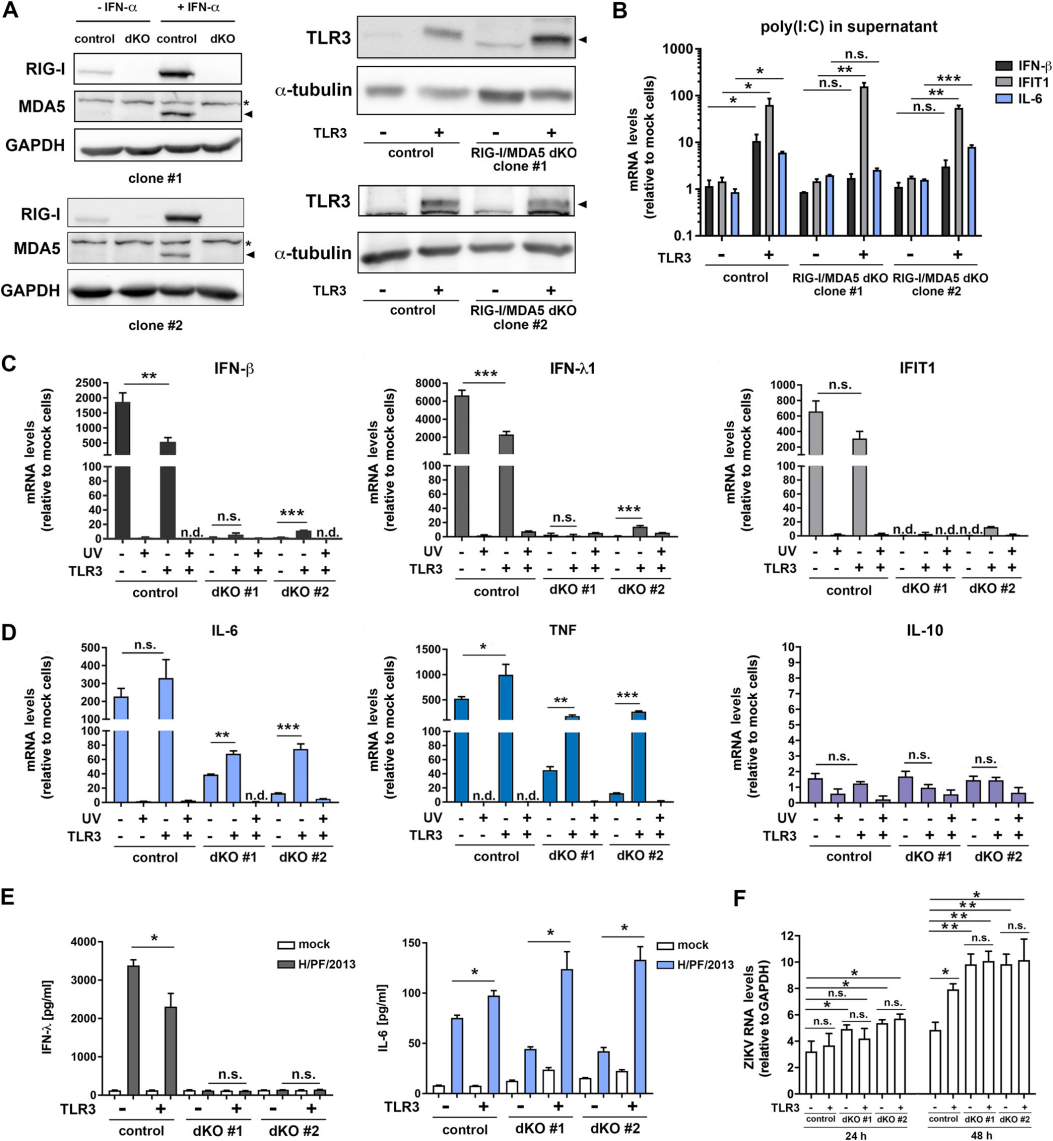
TLR3 activation by ZIKV infection dampens the antiviral response in human epithelial-like cells but enhances the expression of proinflammatory cytokines. (A) (Left) Validation of RIG-I and MDA5 double-knockout (dKO) efficiency in A549 cells treated with 100 U/ml of IFN-α to confirm the absence of MDA5 under MDA5-inducing conditions. The asterisk marks unspecific cross-reaction of the MDA5-specific antibody; the arrowhead points to MDA5. GAPDH served as a loading control. (Right) Expression of TLR3 in control A549 cells or two different A549 cell clones with a RIG-I/MDA5 dKO (clones 1 and 2), reconstituted or not to stably express TLR3, was determined by using Western blotting. α-Tubulin served as a loading control. The black arrowhead indicates the band specific to TLR3. (B) To validate functionality of expressed TLR3, cells were stimulated by adding 10 μg/ml of poly(I·C) into the culture supernatants. After 24 h, samples were collected and induction of IFN-β, IFIT1, and IL-6 expression was determined by RT-qPCR. Values were normalized to GAPDH and to the nonstimulated control, which was set to 1. (C and D) Cells specified below each panel were infected with ZIKV or UV-inactivated virus (strain H/PF/2013; MOI = 1) and 24 h later, harvested by lysis. Amounts of RNAs specified at the top of each graph (antiviral-related pathway [C] and inflammatory [D] cytokines) were quantified by RT-qPCR. (E) IFN-λ and IL-6 released into culture supernatants of infected cells were quantified by ELISA. (F) Viral RNA was quantified by RT-qPCR using a TaqMan probe 24 and 48 h after infection. Values were normalized to GAPDH. The means ± SD from three independent experiments are shown. Statistical significance was assessed by unpaired *t* test with Welch’s correction in comparison to the respective cells indicated by the line. ***, *P* ≤ 0.001; **, *P* ≤ 0.01; *, *P* ≤ 0.05. n.d., not detected.

With the aim to dissect the cross talk between ZIKV-induced RLR and TLR3 cytokine responses, we generated two A549-derived cell clones with RIG-I and MDA5 double knockout (dKO) (Fig. 7A, left). At first, we determined TLR3 expression in these cells and found that they expressed negligible, if any, detectable amounts of TLR3 (Fig. 7A, right), even upon infection with ZIKV (see below). Consistently, these cells were incapable of mounting a detectable cytokine response upon addition of poly(I·C) into the culture medium (Fig. 7B). Therefore, we reconstituted TLR3 expression in both regular A549 control cells and the two dKO cell clones and verified functionality of the cell lines by stimulating them with 10 μg/ml of poly(I·C) added to the culture medium. As shown in Fig. 7B, the antiviral and inflammatory cytokine response was reconstituted (Fig. 7B). Next, we investigated the impact of TLR3 on the IFN response induced by ZIKV by measuring the abundance of antiviral and proinflammatory cytokine mRNAs 24 h postinfection. Consistent with our results in hNPCs and astrocytes, we observed a reduction of the IFN response by functional TLR3 (∼3-fold decrease of IFN-β, IFN-λ1, and IFIT1 [Fig. 7C]). Activation of the IFN response depended on RLRs and was not reconstituted by TLR3 expression in dKO cells (Fig. 7C). Activation required viral replication and was not observed upon inoculation of the cells with UV-inactivated ZIKV (Fig. 7C). In contrast to the suppressive effect of TLR3 on the IFN response, the inflammatory cytokine response, notably TNF, was increased by TLR3 and also in this case required viral replication (Fig. 7D). Amounts of IFN-λ and IL-6 released into culture supernatants as determined by ELISA correlated with mRNA quantifications (Fig. 7E). In line with the reduced production of IFN-λ in cells expressing TLR3, we observed significantly increased ZIKV replication in these cells 48 h after infection (Fig. 7F), i.e., at a time point when enhanced viral replication also became detectable in hNPCs and human astrocytes (Fig. 2D and 5E, respectively). This TLR3-dependent effect was lost in cells that did not express RIG-I and MDA5, while in these cells ZIKV RNA replication was higher than in control cells expressing these two PRRs (Fig. 7F).

### IL-6-dependent activation of STAT3 inhibits IFN signaling.

**FIG 8:**
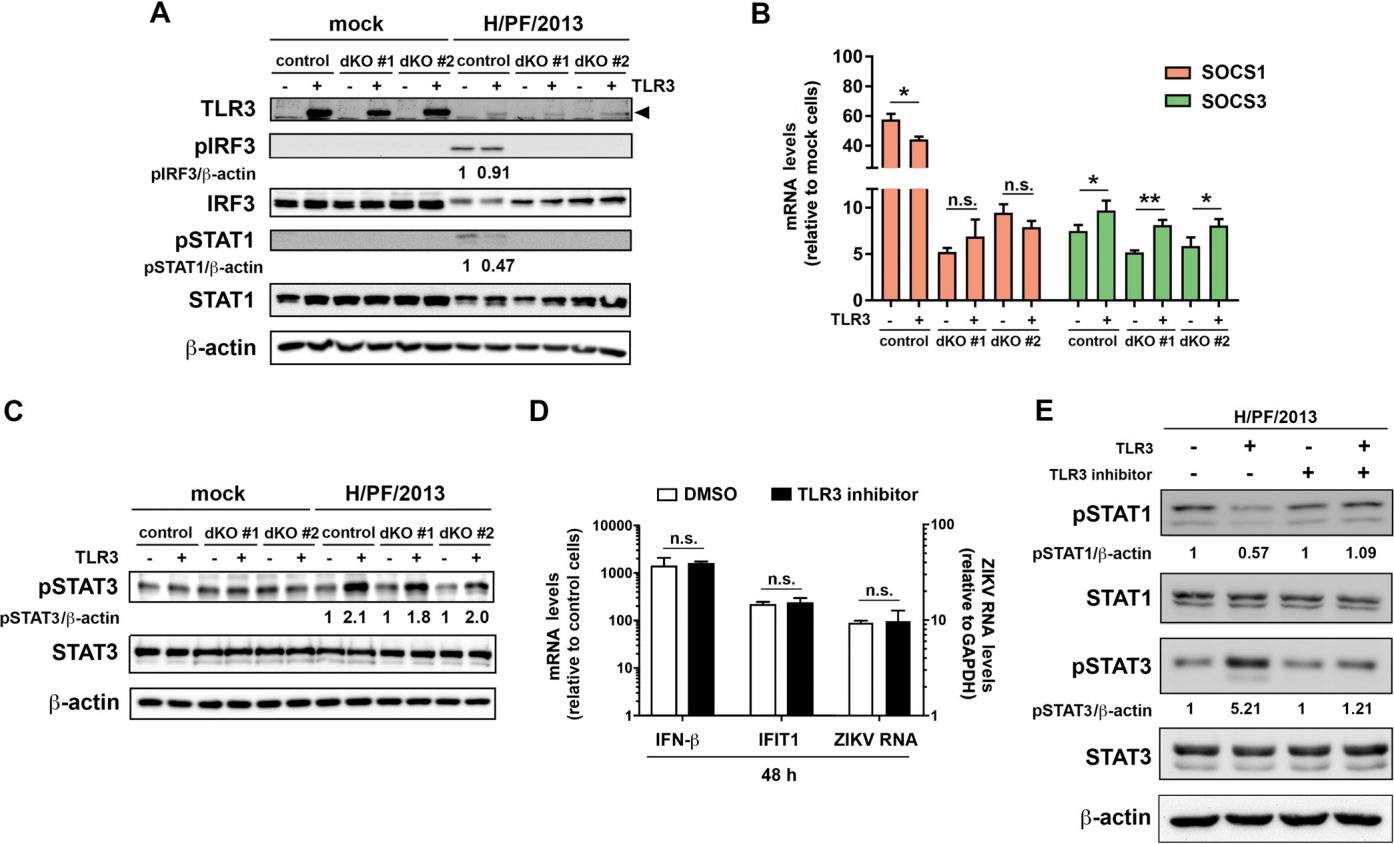
TLR3 activation triggers the STAT3 pathway. (A) The amounts of activated transcription factors IRF3 (pIRF3) and STAT1 (pSTAT1) in two A549 RIG-I and MDA5 dKO cell clones and control A549 cells expressing or not TLR3 were determined by Western blotting 24 h after infection with ZIKV strain H/PF/2013 (MOI = 1). (B) Quantification of SOCS1 and SOCS3 mRNA by RT-qPCR in cells specified at the bottom. (C) Detection of total and phosphorylated STAT3 by Western blotting in lysates of mock- or ZIKV-infected cells. β-Actin was used as a loading control. Numbers below the lanes in panels A and C correspond to protein amounts normalized to β-actin and to control cells, which were set to 1. (D) A549 cells expressing barely detectable levels of TLR3 and unable to mount a TLR3 response were incubated for 1 h with DMSO (control) or a 25 μM concentration of the TLR3/dsRNA complex inhibitor thiophene-carboxamidopropionate, followed by ZIKV infection (strain H/PF/2013; MOI = 5). After 48 h, cells were lysed, total RNA was extracted, and amounts of cytokine mRNA or ZIKV RNA were determined by RT-qPCR. Values were normalized to GAPDH and the respective mock sample (IFN-β and IFIT1) or only to GAPDH (ZIKV RNA). Means ± SD from two independent experiments are shown. Statistical significance was assessed by unpaired *t* test with Welch’s correction. (E) Control A549 cells or cells expressing TLR3 were pretreated like for panel D, and 24 h after ZIKV infection, protein amounts of phosphorylated and total STAT1 and STAT3 were determined by Western blotting. β-Actin was used as a loading control. Numbers below the lanes refer to protein amounts normalized to β-actin and to control cells, which were set to 1.

To gain insight into the mechanism of the observed inhibition of IFN signaling by TLR3, we investigated the activation of key components of this pathway, i.e., IRF3 and STAT1. In A549 cells lacking RIG-I and MDA5, there was no activation of IRF3 or STAT1, regardless of TLR3 (Fig. 8A), which is in line with the lack of IFN expression in these cells (Fig. 7C). In cells stably expressing TLR3, activation of IRF3 was not substantially affected; however, we observed an ∼50% reduction of STAT1 phosphorylation during ZIKV infection (Fig. 8A). To examine whether decreased activation of STAT1 was caused by the SOCS proteins (reviewed in reference 36), we investigated the expression of SOCS1 and SOCS3 in cells infected with ZIKV. SOCS1 expression was significantly decreased in control cells expressing TLR3, whereas in cells devoid of RIG-I and MDA5, there was no effect (Fig. 8B). In the case of SOCS3, there was a consistent elevation of SOCS3 mRNA in the presence of TLR3 (Fig. 8B), correlating with an ∼2-fold-higher activation of STAT3 in all TLR3-expressing cell lines (Fig. 8C).

To corroborate these results with an alternative experimental approach, we employed the dsRNA/TLR3 inhibitor. This compound was specific, as it did not affect IFN-β or IFIT1 expression, thus excluding interference with the RLR pathway (Fig. 8D). Consistently, the inhibitor had no effect on ZIKV RNA replication (Fig. 8D). Next, we determined the impact of this compound on the IFN and the proinflammatory cytokine responses. A549 cells expressing or not TLR3 were infected with ZIKV and 24 h later, cells were treated with the TLR3 inhibitor or left untreated. Pathway activation was determined by analyzing phosphorylation of STAT1 and STAT3. In cells expressing TLR3, pSTAT1 level was reduced and pSTAT3 level was increased compared to those in control A549 cells (Fig. 8E), correlating with the reduction of the IFN response and the increase of the inflammatory response (Fig. 7C to E). In summary, these results obtained with A549 cells confirm those obtained with astrocytes (Fig. 5F), and they support our notion of negative cross talk between TLR3 and RLR signaling contributing to suppression of the ZIKV-induced IFN response.

**FIG 9:**
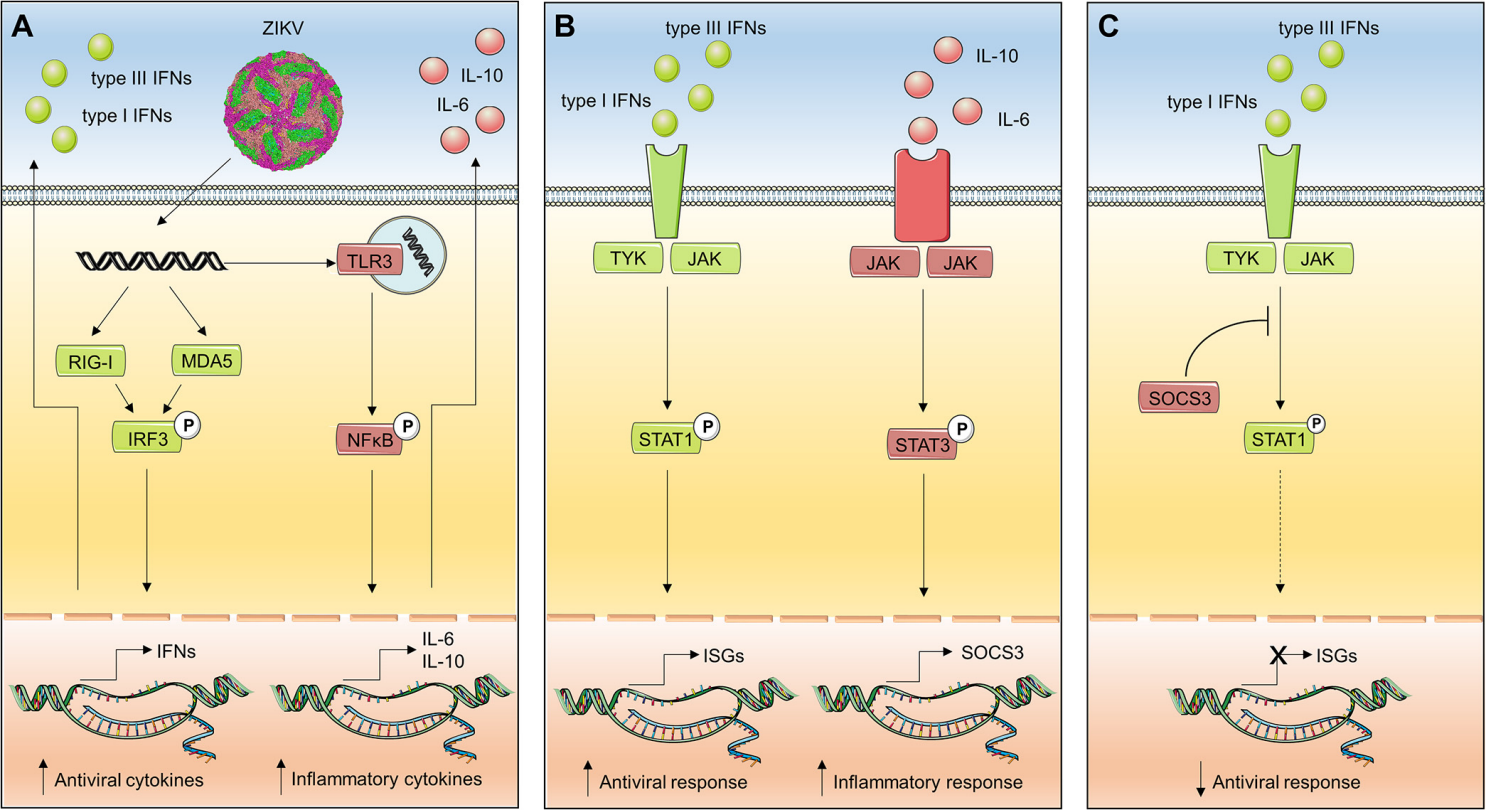
Model for how ZIKV-mediated activation of TLR3 might suppress the IFN response. (A) In the infected cell, ZIKV RNA is sensed by RIG-I-like receptors and TLR3. The RIG-I/MDA5 signaling pathway leads to the production of interferons (IFNs) and, via NF-κB, inflammatory cytokines, notably IL-10 and IL-6. (B) IFNs activate Jak/STAT1 signaling, which turns on the expression of IFN-stimulated genes (ISGs), while IL-6 and IL-10 trigger a signaling cascade resulting in phosphorylation of STAT3 and transcription of STAT3-regulated genes, including SOCS3. (C) SOCS3 inhibits phosphorylation of STAT1 (as suggested in reference 90), thus causing a downregulation of the ISG response. For reasons of simplicity, in this model we only show factors of relevance to this study. The flavivirus virion image was obtained from Protein Data Bank in Europe (PDBE), and the figure was created using modified Smart Servier Medical Art images, which are licensed under a Creative Commons Attribution 3.0 Unported License.

**FIG 10:**
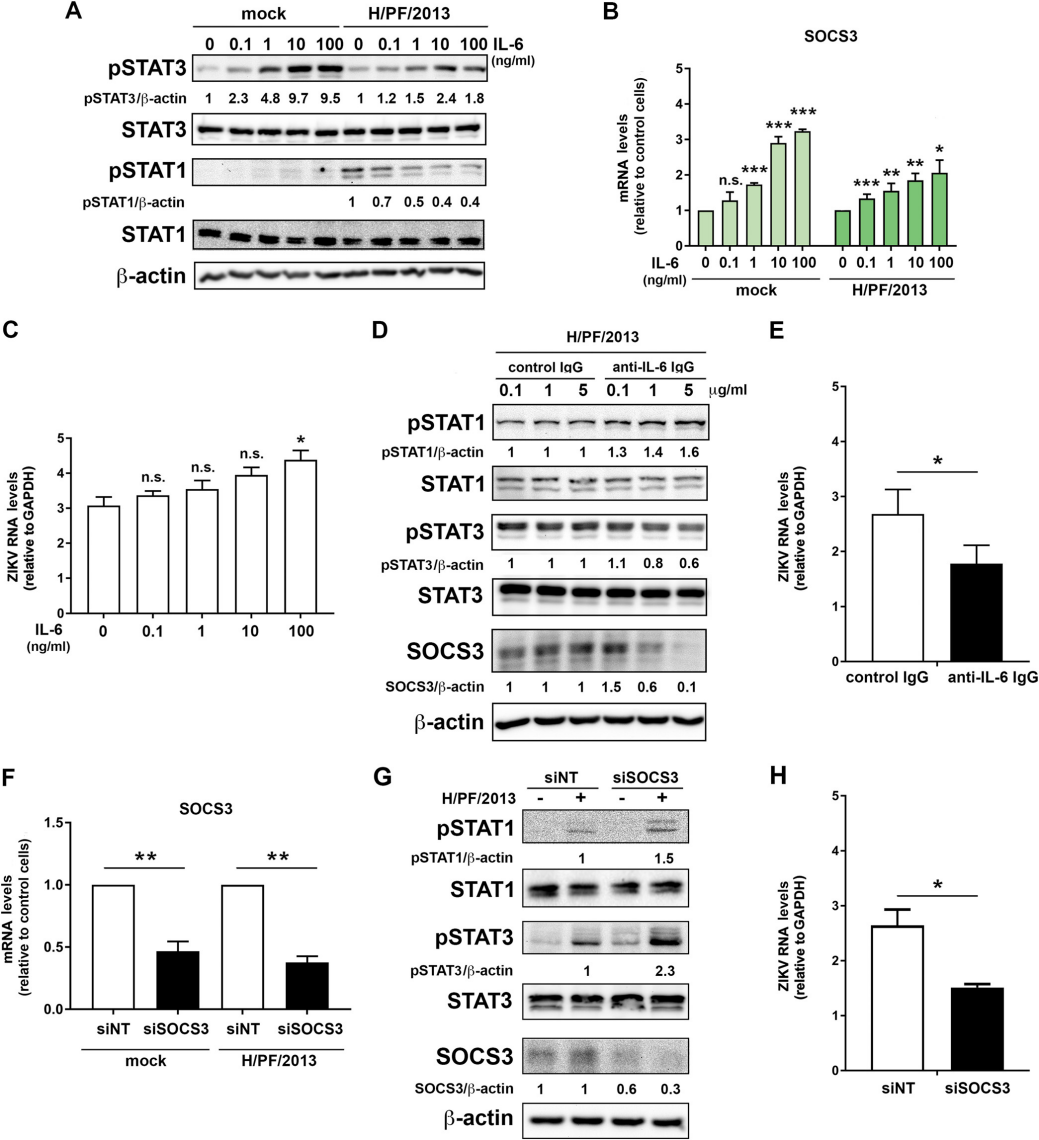
Upregulation of IL-6 activates the STAT3 pathway, leading to a decrease of STAT1 phosphorylation in a SOCS3-dependent manner. (A) Control A549 cells were stimulated with different concentrations of IL-6 1 h prior to and during ZIKV infection. Sixteen hours later, samples were collected and subjected to Western blotting. (B) Quantification of SOCS3 mRNA by RT-qPCR. Values were normalized to GAPDH and to the respective control sample. (C) Total RNA was extracted from the cell lysates, and viral RNA was quantified by RT-qPCR using ZIKV-specific primers and a TaqMan probe. Values were normalized to GAPDH. (D and E) A549 cells expressing TLR3 were infected with ZIKV in the presence of 0.1, 1, or 5 μg/ml of either anti-IL-6 IgG or control IgG. (D) After 24 h, expression of STATs was determined by Western blotting. (E) Total RNA was extracted from cell lysates, and RNA amounts were quantified by RT-qPCR using ZIKV-specific primers and a TaqMan probe. Values were normalized to GAPDH. (F to H) A549 cells overexpressing TLR3 were transfected with nontargeting or SOCS3-specific siRNA for 48 h and subsequently infected with ZIKV for 24 h. Expression of SOCS3 was determined by RT-qPCR (F) and Western blotting (G), along with phosphorylated and total protein levels of STAT1 and STAT3. (H) ZIKV RNA amounts were quantified as for panel E. (A, D, and G) Numbers below the lanes correspond to protein amounts relative to β-actin, which was set to 1 for respective control cells. For all PCR-based analyses, mean values ± SD from three independent experiments are shown. Statistical significance was assessed by unpaired *t* test with Welch’s correction. ***, *P* ≤ 0.001; **, *P* ≤ 0.01; *, *P* ≤ 0.05.

Given the observed increase of STAT3 phosphorylation (Fig. 8C) and the upregulation of IL-6 production in cells expressing TLR3 (Fig. 7E), we hypothesized that TLR3-induced IL-6 production might induce SOCS3 expression, which, in turn, would impair the IFN response via suppression of STAT1 phosphorylation (Fig. 9). To address this assumption, we stimulated control A549 cells with different concentrations of IL-6 prior to ZIKV infection, with the lowest concentration corresponding to IL-6 amounts released upon ZIKV infection (Fig. 7E). We observed an IL-6 dose-dependent reduction of STAT1 phosphorylation and an increase of STAT3 phosphorylation (Fig. 10A). Although this increase was more pronounced in control A549 cells, it was still well detectable in ZIKV-infected cells. In line with this differential activation, we observed a significant dose-related increase of SOCS3 expression in control and ZIKV-infected cells (Fig. 10B). While there was a correlating trend of increased ZIKV replication, possibly due to inhibition of STAT1 activation, this trend reached statistical significance only for the highest IL-6 dose (Fig. 10C). This result suggests that even small amounts of secreted IFN are capable of suppressing the virus.

To validate that IL-6 was also responsible for the observed signaling pathway alterations in ZIKV infected cells, we added an IL-6-neutralizing antibody into the culture supernatant of infected cells. We observed a dose-dependent increase of pSTAT1, correlating inversely with a downregulation of STAT3 phosphorylation and reduced SOCS3 protein levels in infected cells (Fig. 10D). In agreement with our results obtained with astrocytes, the observed increase in pSTAT1 levels, and, hence, IFN pathway activation, correlated with a significant decrease of ZIKV replication (Fig. 10E). Finally, we lowered SOCS3 expression by using siRNAs, achieving an ∼60% reduction of mRNA (Fig. 10F) and a 40 to 70% decrease in the protein amount (Fig. 10G). Under these conditions, we observed markedly increased phosphorylation of STAT1 and STAT3 (Fig. 10G) and significantly decreased replication of ZIKV (Fig. 10H). Based on these results, we hypothesize that TLR3 stimulation by ZIKV upregulates IL-6 and IL-10 production, with these cytokines promoting STAT3-mediated SOCS3 expression. Elevated SOCS3 levels inhibit the JAK/STAT signaling axis required to mount the IFN response (Fig. 9), thus increasing ZIKV replication.

## DISCUSSION

In this study, we showed that ZIKV induces a robust antiviral response, both in human epithelial cells and in human iPSC-derived astrocytes but not in hNPCs. In the developing brain, ZIKV was shown to preferentially infect hNPCs (9), consistent with our studies demonstrating high levels of infection and virus replication in these cells. Interestingly, hNPCs did not mount a significant IFN response to ZIKV, in agreement with previous studies by Hanners and colleagues (40). We observed only a marginal, delayed ISG response, which is consistent with a study by Tang and colleagues, who showed delayed transcriptional changes in hNPCs infected with ZIKV (9). However, these cells are not *per se* immunodeficient, as an IFN response was readily induced by SeV infection (a potent inducer of RIG-I signaling) and poly(I·C) transfection into these cells, although the IFN response was much less pronounced than in human iPSC-derived astrocytes and A549 cells. Of note, this result was obtained with two independent hNPC lines, arguing against clonal effects. We note that (i) stem cells do not induce an IFN response to viral infection and are refractory to IFN stimulation; (ii) stem cells intrinsically express specific groups of ISGs, which mediate antiviral resistance; (iii) responsiveness to IFN stimulation develops during differentiation (41); and (iv) hNPCs are differentiated progeny of iPSCs. Therefore, we speculate that low IFN competence of hNPCs might be a reflection of their (partial) stemness. Consistently, it has been reported that ZIKV infection of hNPCs activates, depending on the presence of IRF3 and NF-κB, ISGs but not IFN (42). It is also possible that ZIKV-triggered immune evasion might contribute to this phenotype. For instance, ZIKV can antagonize the IFN response by using the AXL receptor, which triggers a signaling cascade inducing the expression of SOCS1, which, in turn, suppresses IFN signaling via the JAK/STAT pathway (34). Moreover, ZIKV nonstructural proteins have been reported to contribute to evasion of innate antiviral defense in several ways, similar to other flaviviruses as shown in earlier studies (21, 22, 43–45).

Consistent with previous reports (21, 39), the lack of IFN response in hNPCs precluded the identification of the engaged PRRs, and therefore, we employed iPSC-derived astrocytes and A549 epithelial cells. By using knockdown and KO approaches, we found that ZIKV is sensed mainly by RIG-I in A549 cells, whereas in human astrocytes, RIG-I and MDA5 were involved in ZIKV recognition. This result is in line with a recent report based on human trophoblasts, in which KO of either RIG-I or MDA5 resulted in strong reduction of IFN-β expression (22). In the case of Japanese encephalitis virus (JEV), RIG-I was reported to limit replication in the brain (46–48); however, other flaviviruses, such as West Nile virus (WNV) and dengue virus (DENV), were shown to be sensed by both RIG-I and MDA5 (49–52). During WNV infection of mouse embryonic fibroblasts, viral RNA is sensed by RIG-I during early time points after infection (<12 h), whereas at later time points (>24 h), sensing is mediated by MDA5, which jointly with RIG-I contributes to the antiviral response (52). Whether such a temporal dependency might also apply to ZIKV-infected human iPSC-derived astrocytes remains to be determined.

Multiple studies have shown that in addition to RLRs, TLR3 is a potent inducer of immune responses against flaviviruses (34, 51, 53, 54). The involvement of TLR3 in ZIKV recognition was first suggested by Hamel and coworkers, who used human skin fibroblasts and observed a strong increase of viral RNA replication in cells depleted of TLR3, however, without influencing type I IFN mRNA production (7). In addition, Dang and colleagues showed that in cerebral organoids, ZIKV activation of TLR3 induces apoptosis and impairs neurogenesis (24). In this study, we used hNPCs and astrocytes and detected upregulation of TLR3 levels after ZIKV infection, consistent with previous studies using poly(I·C) (28) or bacterial or viral infection (29, 30). Several studies showed that TLR3 expression can be upregulated in response to type I IFNs (55–57); however, we did not observe a significant increase of IFN-α or IFN-β production in astrocytes and A549 cells infected with ZIKV, arguing for another mechanism of TLR3 induction.

By using A549 cells devoid of RIG-I/MDA5 and stably expressing TLR3, we did not detect significant IFN expression upon activation of TLR3 by exogenously added poly(I·C) or infection with ZIKV. This observation is comparable to our earlier results obtained with primary human hepatocytes and human hepatoma cells stably expressing TLR3 (58). Strikingly, activation of TLR3 by ZIKV even decreased the induction of type I and III IFN expression, concomitant with increased expression of proinflammatory cytokines, such as IL-6 and TNF. This result is reminiscent of observations made by Le Goffic and colleagues, who reported that upon infection of human pulmonary epithelial cells with influenza A virus, activation of both RIG-I and TLR3 induced a proinflammatory response, while type I IFN was triggered mainly by RIG-I (25). Along the same lines, it has been reported that TLR3 activation upon infection of hepatocytes with hepatitis C virus (HCV) only moderately induced ISGs but profoundly induced the production of chemokines and inflammatory cytokines (26, 59).

The endosomal localization of TLR3 raises the question of how ZIKV, replicating in confined replication organelles within the cytoplasm, is sensed. Our experiments with caspase inhibitors suggest that viral dsRNA rather than apoptotic cell debris induces the signaling cascade (A. Plociennikowska and R. Bartenschlager, unpublished data). Since in unstimulated cells TLR3 resides at membranes of the endoplasmic reticulum, which is the site from which viral replication organelles are derived (60), we speculate that the endoplasmic reticulum might be a site of interaction between TLR3 and ZIKV RNA.

The observation that TLR3 activation by ZIKV induces proinflammatory cytokines at the expense of the IFN response, along with data showing robust activation of a TLR3-dependent immune response in hNPCs after poly(I·C) stimulation (40), led us to investigate whether TLR3 signaling might suppress the IFN response upon ZIKV infection in neural progenitor cells. Indeed, we found that pharmacological inhibition of TLR3 in hNPCs increased IFN and IFIT1 production and abolished IL-10 and TNF expression. Of note, elevation of the IFN response upon inhibition of TLR3 was also observed in human iPSC-derived astrocytes (concomitant with reduced ZIKV replication) and A549 cells. This decrease of viral RNA and inflammatory cytokines upon TLR3 inhibition is in agreement with a recent study that employed human astrocytes and microglial cells (23). However, these authors also reported that TLR3 inhibition in astrocytes caused a significant decrease of IFN-β, RANTES, and IP-10 secretion after infection with a Puerto Rican strain of ZIKV. Although in that and several other studies differences regarding virulence and immune responses have been reported for different ZIKV strains (33, 39, 61, 62), we did not observe profound differences between the prototypic MR766 African strain and the Asian H/PF/2013 strain (Plociennikowska and Bartenschlager, unpublished).

TLR3 and RLR signaling is induced by receptors residing in different cell compartments, making competition for viral RNA binding unlikely. Hence, we speculate that the regulation is mediated by autocrine/paracrine signaling of secreted cytokines. In support of a paracrine effect, we found that inhibition of Jak/STAT signaling by ruxolitinib abolished that TLR3 inhibitor effect. Several groups reported that proinflammatory cytokines, e.g., IL-6 or TNF, and type I IFN mutually affect their signaling pathways (63–67). Consistently, Hotz and colleagues demonstrated that priming of endosomal TLR pathways by poly(I·C) induced tolerance toward RLR stimulation (27). Moreover, Livengood and colleagues described elevation of TLR3-dependent production of proinflammatory cytokines and chemokines in RIG-I knockdown cells (68). Our observation of increased STAT3 phosphorylation upon ZIKV infection in the presence of TLR3 and the downregulation of STAT1 signaling (Fig. 9) is in agreement with the well-established interplay between these two transcription factors (recently reviewed in reference 69). We speculate that in our case the negative STAT3 feedback may act through SOCS3-mediated interference of STAT1 activation, as described earlier (70, 71). In turn, this downregulates the expression of multiple ISGs, including the RLRs that sense ZIKV (RIG-I and MDA5) as well as IFN-λ itself (72). Additionally, Lu and colleagues demonstrated that STAT3 negatively regulates the expression of IRF7, IRF9, STAT1, and STAT2 (73), which might affect the induction of IFNs independent of SOCS3. However, further studies of the STAT1/STAT3 interplay in ZIKV-infected cells by using pharmacological approaches will be difficult given the often insufficient specificity of STAT3 inhibitors such as Stattic (74).

Using hNPCs, we were not able to detect production and release of IL-6 upon ZIKV infection. However, STAT3/SOCS3 activation is not limited to induction by IL-6, as several other IL-6-like cytokines as well as anti-inflammatory IL-10 family members and growth factors were shown to induce STAT3 signaling in different cell lines (reviewed in references 75 to 77). The expression of IL-10 might explain why, despite low or absent amounts of IL-6, we still observed an increase in IFN signaling upon TLR3 inhibition in hNPCs. However, also other TLR3-mediated factors might play a role in these cells, especially since the stemness state of progenitor cells differentiates their innate immune responses from that of other cells (41). Hence, while our observations support an antagonistic effect of TLR3- onto RLR-induced signaling, the exact molecular mechanism by which TLR3 activation interferes with the IFN response during ZIKV infection in hNPCs remains to be elucidated.

TLR3 is expressed to high levels during early stages of neurogenesis, but abundance declines with ensuing development. Moreover, TLR3 activation reduces cell proliferation and neurosphere formation (78). Dang and colleagues demonstrated that ZIKV depletes neural progenitor cells in human cerebral organoids in a TLR3-dependent manner (24). In addition, ZIKV infection increases the amounts of neurotoxic cytokines, i.e., TNF and IL-1β, in neuronal cultures and blockade of these factors suppresses neuronal cell death (79). In line with our results, these data suggest that TLR3 activation by ZIKV might be an important contributor to neuronal defects in the developing brain. Two recent studies identified monocytes as the main target of ZIKV infection, with infected monocytes possibly acting as a Trojan horse to facilitate ZIKV infiltration of the brain (80, 81). Both groups observed enhanced expression of inflammatory cytokines but only low expression of ISGs, reminiscent of the TLR3 activation we report here.

In conclusion, we found that ZIKV activates TLR3 to produce an inflammatory response, which likely dampens the antiviral response induced by RIG-like receptors.

## MATERIALS AND METHODS

### Cell lines.

The A549, 293T, and VeroE6 cell lines were cultured in Dulbecco’s modified Eagle medium (DMEM; Life Technologies, Germany) supplemented with 10% fetal calf serum (GE Healthcare, Germany), 100 μg/ml of penicillin, 100 μg/ml of streptomycin (Sigma-Aldrich, Germany), and nonessential amino acids (Life Technologies). Generation of hNPCs was described earlier (60). Cells were cultured on dishes coated with 0.1 mg/ml of polyornithine and 10 μg/ml of laminin (both from Sigma-Aldrich) in DMEM/F-12 containing 2 mM GlutaMAX, 1% N-2 (catalog no. 17502), 2% B-27 (catalog no. 17504), 50 μM β-mercaptoethanol, and 10 ng/ml of epidermal growth factor (EGF) (all from Gibco, Life Technologies), as well as 10 ng/ml of fibroblast growth factor 2 (FGF2; PeproTech) and 50 μg/ml of penicillin and 50 μg/ml of streptomycin (Sigma-Aldrich, Germany). Generation of human iPSC-derived astrocytes was performed according to a previously published protocol (82), with slight modifications. In brief, hNPCs were maintained at high density, grown on Matrigel (BD Bioscience) in NPC medium (DMEM/F-12 supplemented with 1× N-2, 1× B-27-RA [Invitrogen], 20 ng/ml of FGF2 [PeproTech], and 20 ng/ml of ciliary neurotrophic factor [CNTF; PeproTech]) and split approximately 1:3 to 1:4 every week by using Accutase (Merck Millipore, Germany). NPCs were differentiated into astrocytes by seeding single-cell suspensions at a density of 15,000 cells/cm^2^ on Matrigel-coated plates in astrocyte medium (ScienCell; catalog no. 1801-b), 2% fetal bovine serum (FBS; ScienCell; catalog no. 0010), astrocyte growth supplement (ScienCell; catalog no. 1852), and 10 U/ml of penicillin/streptomycin solution (ScienCell; catalog no. 0503). Alternatively, human iPSC-derived astrocytes were generated according to the transcription factor-based programming protocol (83), with modifications. In brief, iPSCs were seeded on Matrigel-coated plates at a density of 62,500 cells/cm^2^ per well in mTeSR medium, supplemented with a Rho kinase inhibitor (both from STEMCELL Technologies, Canada). Subsequently, cells were transduced with lentiviruses encoding *rtTa*, *Sox9*, and *Nfib* and maintained in expansion medium (DMEM/F-12 containing 10% FBS, 1% N-2 supplement, and 1% GlutaMAX [all from Thermo Fisher Scientific]) supplemented with 2.5 μg/ml of doxycycline (Sigma-Aldrich) and 1.25 μg/ml of puromycin and 200 μg/ml of hygromycin (both from Thermo Fisher Scientific). From day 3 onwards, cells were cultured in expansion medium with gradual addition of FGF medium (neurobasal medium supplemented with 2% B-27, 1% nonessential amino acids [NEAA], 1% GlutaMAX, and 1% FBS [all from Thermo Fisher Scientific] and 8 ng/ml of FGF, 5 ng/ml of CNTF, and 10 ng/ml of BMP4 [all from PeproTech]), with 2.5 μg/ml of doxycycline and 200 μg/ml of hygromycin, until expansion medium was completely replaced with FGF medium with 2.5 μg/ml of doxycycline. Finally, medium was replaced with B-27-supplemented final medium (neurobasal A medium, 2% B-27, 1% GlutaMAX, 5% FBS) containing 2.5 μg/ml of doxycycline. All cell cultures were maintained in a humidified incubator with 5% CO_2_ at 37°C.

### Stable cell lines.

Cell lines were generated by transduction using lentiviral particles produced in 293T cells. Briefly, cells were transfected with the packaging plasmids pCMV-gag-pol and pMD2-VSV-G (kind gifts from Didier Trono) and the pWPI vector carrying the gene of interest by using the calcium phosphate method. Supernatants were collected 48, 56, and 72 h after transfection, filtered through a 0.45-μm-pore-size filter, and used to transduce target cells. Transduced cells were selected by culturing them in medium containing 1 μg/ml of puromycin unless otherwise stated.

A549 cells with a RIG-I knockout (KO) were generated using the single guide RNA (sgRNA) 5′-CTGTTGGAGCTCCAGGAGGA-3′ inserted into the lenti-CRISPR plasmid encoding the Cas9 nuclease (Addgene; catalog no. 52961) and lentiviruses were produced as described above. The A549-derived RIG-I KO single-cell clone was described earlier (84). MDA5 KO cell lines were generated using the following sgRNAs: 5′-GGATTGTGCAGAAAGAAAAC-3′ (no. 1) and 5′-AATCAGAGCCTGTTAACTCT-3′ (no. 2). MAVS KO cells were generated in the analogous manner using the protocol described earlier (85) and the sgRNA 5′-TCAGCCCTCTGACCTCCAGCG-3′. Single-cell colonies were tested for double KO using Western blotting and functional assays. The sgRNA used to generate A549 RIG-I KO cells and sgRNA no. 2 targeting MDA5 were used for CRISPR/Cas9-mediated KO in human iPSC-derived astrocytes. Cell lines were generated using lentiviral transduction at the beginning of the cellular differentiation process in the presence of 8 μg/ml of Polybrene (Merck Millipore, Germany). Transduced cells were selected with 4 μg/ml of puromycin. To achieve low or high RIG-I expression levels in A549-derived RIG-I KO cells, we used pWPI vectors containing a ROSA26 or an EF1-alpha promoter, respectively. For TLR3 stable expression, we used a pWPI vector with a ROSA26 promoter. Cells were selected in medium containing 5 μg/ml of blasticidin.

### RNA interference.

A549 cells were transfected with 10 nM SMARTpool siRNAs targeting human *SOCS3* or control nontargeting siRNA (Dharmacon, USA) using Lipofectamine RNAiMAX reagent (Invitrogen, USA) according to the protocol of the manufacturer. All transfections were carried out in Opti-MEM medium (Invitrogen), which was replaced after 8 h with standard culture medium described above. SOCS3 mRNA and protein levels were determined 72 h after transfection.

### Viruses.

The ZIKV strain H/PF/2013 was obtained from European Virus Archive Global (EVAg; France). Virus stocks were prepared by virus amplification in VeroE6 cells. Supernatants of infected cell cultures or medium from uninfected cells were harvested from day 3 to day 7 postinfection. Supernatants were filtered through a 0.45-μm-pore-size filter and stored at −80°C. For infection of hNPCs and lentiviral transduction of differentiating astrocytes, viruses were purified by sedimentation onto a 20% sucrose cushion by 3 h of centrifugation at 28,000 rpm (SW32 Ti rotor and Optima LE-80K ultracentrifuge [Beckman]). Sendai virus (SeV) and Mengo virus Zn mutant (MnZnV) were kindly provided by R. Zawatzky (German Cancer Research Center, Heidelberg, Germany) and by F. van Kuppeveld (Utrecht University, Utrecht, The Netherlands), respectively. In all experiments, inocula were replaced with fresh cell culture medium 2 h postinfection.

### Plaque assays.

Confluent monolayers of VeroE6 cells were infected with serial 10-fold dilutions of virus-containing culture supernatants for 2 h at 37°C. Inocula were removed and replaced with serum-free MEM (Gibco, Life Technologies) containing 1.5% carboxymethylcellulose (Sigma-Aldrich). Four days postinfection, cells were fixed for 2 h at room temperature with formaldehyde directly added to the medium at a final concentration of 5%. Fixed cells were washed extensively with water and stained with a solution containing 1% crystal violet and 10% ethanol for 30 min. After rinsing with water, the number of plaques was counted and virus titers were calculated.

### Reagents.

For stimulation of RIG-like receptor-dependent signaling pathways, cells were mock transfected or transfected with 100 ng/ml of poly(I·C) (Sigma-Aldrich, Germany) using Lipofectamine 2000 (Life Technologies, Germany) according to the manufacturer’s protocol. TLR3 was activated by addition of poly(I·C) into the cell culture supernatant (“feeding”) using a final concentration of either 10 or 50 μg/ml. One hour prior to infection/stimulation of cells, the TLR3/dsRNA complex inhibitor thiophene-carboxamidopropionate (Merck Millipore, Germany) was added into the cell culture supernatant to a final concentration of 25 μM. Ruxolitinib (Cayman Chemical, USA), a Janus-associated kinase (JAK) inhibitor, was added into the cell culture supernatant at a final concentration of 1 μM 2 h prior to infection. Both inhibitors were present during the complete course of the experiment. Cells cultured in medium containing the matching concentration of DMSO served as a control. Activation of STAT3 was achieved by stimulation of cells with the indicated concentrations of recombinant human IL-6 (PeproTech, Germany) starting 1 h prior to infection until the end of the experiment. Neutralizing monoclonal antibody against human IL-6 (mouse IgG1) and isotype control for mouse IgG1 (both from InvivoGen, USA) were added into the cell culture supernatant at a concentration of 0.1 to 5 μg/ml and were present during the complete course of the experiment. To activate expression of IFN-stimulated genes (ISGs), cells were treated with either 100 or 250 U/ml of recombinant IFN-α (PBL, USA).

### Quantitative RT-PCR.

Total cellular RNA was isolated from 1 × 10^5^ cells, seeded into each well of a 24-well plate, by using the NucleoSpin RNA Plus extraction kit (Macherey-Nagel, Germany) according to the manufacturer’s protocol. RNA was reverse transcribed into cDNA using a high-capacity cDNA reverse transcription kit (Applied Biosystems) and used for real-time PCR with the iTaq Universal SYBR green Supermix (Bio-Rad, Germany). Glyceraldehyde-3-phosphate dehydrogenase (GAPDH) mRNA amounts were measured in parallel and used for normalization of input RNA.

ZIKV RNA was quantified using the qSCRIPT XLT 1-Step RT-qPCR ToughMix (Quantabio, USA) using a TaqMan probe as described earlier (86). GAPDH mRNA was measured in the same way. Sequences of used primers and probes are given in Table 1; primers and probes were purchased from Sigma-Aldrich, Germany. RT-qPCRs were performed using a C1000 Touch thermal cycler (CFX96; Bio-Rad, Germany). Data were analyzed by using the Δ*CT* or ΔΔ*CT* method (87).

**TABLE 1:** Sequences of primers and TaqMan probes used for quantitative RT-PCR

Primer or probe	Primer sequence (5′–3′)^*a*^	
	Forward	Reverse
Primers
GAPDH	GAAGGTGAAGGTCGGAGTC	GAAGATGGTGATGGGATTTC
IFIT1	GAAGCAGGCAATCACAGAAA	TGAAACCGACCATAGTGGAA
MX1	AGCCACTGGACTGACGACTT	GAGGGCTGAAAATCCCTTTC
IFN-β	CGCCGCATTGACCATCTA	GACATTAGCCAGGAGGTTCTC
IL-29	CGCCTTGGAAGAGTCACTCA	GAAGCCTCAGGTCCCAATTC
RIG-I	CCCTGGTTTAGGGAGGAAGA	TCCCAACTTTCAATGGCTTC
MDA5	TCGTCAAACAGGAAACAATGA	GTTATTCTCCATGCCCCAGA
MAVS	TGGAGTCCTCCTCTGACCTG	GCTGGAAGGAGACAGATGGA
IL-6	ACTCACCTCTTCAGAACGAATTG	CCATCTTTGGAAGGTTCAGGTTG
IL-10	GGGGAGAACCTGAAGACCCT	CGGCCTTGCTCTTGTTTTCA
TNF	GAGGCCAAGCCCTGGTATG	CGGGCCGATTGATCTCAGC
SOCS1	GTCACTGCGCTCCAGTAGAA	TAGGAGGTGCGAGTTCAGGT
SOCS3	CCTGCGCCTCAAGACCTTC	GTCACTGCGCTCCAGTAGAA
ZIKV	CCGCTGCCCAACACAAG	CCACTAACGTTCTTTTGCAGACAT
ZIKV probe	FAM-AGCCTACCTTGACAAGCAATCAGACACTCAA-TAM	
GAPDH probe	HEX-CAAGCTTCCCGTTCTCAGCCT-TAM	

a FAM, 6-carboxyfluorescein; HEX, 6-carboxy-2,4,4,5,7,7-hexachlorofluorescein; TAM, 6-carboxytetramethylrhodamine.

### Immunofluorescence microscopy.

Cells were seeded onto glass coverslips at a density of 0.5 × 10^5^ cells per well of a 24-well plate 1 day prior to the experiment. For immunofluorescence microscopy of hNPCs, coverslips were coated with polyornithine and laminin. Cells were fixed 24, 48, or 72 h postinfection in 100% ice-cold methanol for 10 min, followed by 1 h of incubation in blocking buffer (5% bovine serum albumin [BSA] in phosphate-buffered saline [PBS]). After being washed with PBS, samples were incubated for 2 h with a nestin-specific mouse monoclonal primary antibody (1:500; Merck Millipore, Germany) or a S100β subunit-specific antibody (1:250; s2532; Sigma-Aldrich), each diluted in 3% BSA/PBS. For detection of ZIKV NS3, a cross-reacting rabbit antibody raised against DENV NS3 was used as described earlier (60). After being washed with PBS, samples were incubated in darkness for 1 h with anti-rabbit Alexa Fluor 488- and anti-mouse Alexa Fluor 568-conjugated secondary antibodies (Life Technologies and Invitrogen, respectively). Nuclear DNA was stained with 4′,6-diamidino-2-phenylindole (DAPI; MoBiTec, Germany) for 10 min and after extensive washing with PBS and rinsing with water, coverslips were mounted with Fluoromount G (Southern Biotechnology Associates, USA). Images were acquired with a Nikon Ti Eclipse microscope, and the infection rate was quantified by using the pixel and object classification workflow of the ilastik software package (88). Noninfected and infected cells were counted on the output of the object classification workflow using a threshold-based method in the ImageJ/Fiji software package (89). For visualization purposes, images were cropped in ImageJ/Fiji and the Gamma correction was adjusted to a value of 1.5 in the Adobe Photoshop CS6 software package.

### Western blotting.

Cells seeded at a density of 1 × 10^5^ per well of a 24-well plate were lysed in Laemmli buffer (30 mM Tris [pH 6.8], 0.05% [wt/vol] bromophenol blue, 10% [wt/vol] glycerol, 1% [wt/vol] SDS, 2.5% β-mercaptoethanol) and heated to 95°C for 10 min. Proteins were subjected to SDS-PAGE and blotted onto a polyvinylidene difluoride (PVDF) membrane by semidry blot (Bio-Rad, Germany). Membranes were blocked with either 5% BSA or 5% milk (both in PBS containing 0.1% Tween 20) for 1 h at room temperature, followed by overnight incubation with primary antibodies at 4°C. The Mx1-specific mouse antibody was obtained from Georg Kochs (University of Freiburg, Germany). The following commercially available antibodies were used in addition: rabbit polyclonal anti-MAVS and anti-MDA5 (Enzo Life Sciences, Switzerland), mouse monoclonal anti-RIG-I (AdipoGen Life Sciences, Switzerland), mouse monoclonal anti-TLR3 (Abcam), mouse monoclonal anti-pSTAT1 (Y701) and anti-STAT1 (BD Biosciences, USA), rabbit monoclonal anti-pIRF3 (S396) (Cell Signaling Technology, USA), mouse monoclonal anti-IRF3 (Santa Cruz Biotechnology, USA), rabbit monoclonal anti-pSTAT3 (Y705), anti-STAT3, and anti-SOCS3 (D6E1T) (Cell Signaling Technology), mouse monoclonal anti-GAPDH (Santa Cruz Biotechnology), and mouse monoclonal anti-β-actin and anti-α-tubulin (Sigma-Aldrich, Germany). Anti-mouse and anti-rabbit secondary antibodies coupled to horseradish peroxidase (Sigma-Aldrich) were incubated at room temperature for 1 h. After a washing with PBS containing 0.1% Tween 20, proteins were visualized using the Clarity Western ECL substrate (Bio-Rad, Germany) and a high-sensitivity charge-coupled-device (CCD) camera (ChemoCam Imager 3.2; INTAS, Germany). The ImageJ/Fiji software Gel Analyzer tool was used for quantification of signals that were normalized to the loading control.

### Enzyme-linked immunosorbent assay (ELISA).

Culture supernatants of infected cells were collected and virus contained therein was inactivated with 1% Triton X-100. Amounts of IFN-α (all subtypes) and IFN-λ1-3 (both from PBL, USA) and IL-6 (R&D Systems, USA) were quantified according to the instructions of the manufacturer using a Thermo Multiskan EX microplate photometer (Thermo Fisher Scientific Inc., USA).

### Statistics.

Statistical analysis was performed using the GraphPad Prism v7 software package. Significance was determined using unpaired *t* test with Welch’s correction.
